# A highly predictive signature of cognition and brain atrophy for progression to Alzheimer's dementia

**DOI:** 10.1093/gigascience/giz055

**Published:** 2019-05-11

**Authors:** Angela Tam, Christian Dansereau, Yasser Iturria-Medina, Sebastian Urchs, Pierre Orban, Hanad Sharmarke, John Breitner, Pierre Bellec, Alzheimer's Disease Neuroimaging Initiative

**Affiliations:** 1 Centre de Recherche de l'Institut Universitaire de Gériatrie de Montréal, 4545 chemin Queen-Mary, Montréal, QC, H3W 1W4, Canada; 2 Centre for the Studies on Prevention of Alzheimer's Disease, Douglas Mental Health University Institute Research Centre, 6875 Lasalle Boulevard, Montréal, QC, H4H 1R3, Canada; 3 Montreal Neurological Institute, McGill University, 3801 University Street, Montréal, QC, H3A 2B4, Canada; 4 Département d'informatique et de recherche opérationnelle, Université de Montréal, 2920 chemin de la Tour, Montréal, QC, H3T 1J4, Canada; 5 Centre de Recherche de l'Institut Universitaire en Santé Mentale de Montréal, 7331 rue Hochelaga, Montréal, QC, H1N 3V2, Canada; 6 Département de psychiatrie, Université de Montréal, 2900 boulevard Édouard-Montpetit, Montréal, QC, H3T 1J4, Canada; 7 Department of Psychiatry, McGill University, 1033 Pine Avenue West, Montréal, QC, H3A 1A1, Canada; 8 Département de psychologie, Université de Montréal, 90 avenue Vincent d'Indy, Montréal, QC, H3C 3J7, Canada

**Keywords:** Alzheimer's disease, mild cognitive impairment, machine learning, neuroimaging, cognition

## Abstract

**Background:**

Clinical trials in Alzheimer's disease need to enroll patients whose cognition will decline over time, if left untreated, in order to demonstrate the efficacy of an intervention. Machine learning models used to screen for patients at risk of progression to dementia should therefore favor specificity (detecting only progressors) over sensitivity (detecting all progressors), especially when the prevalence of progressors is low. Here, we explore whether such high-risk patients can be identified using cognitive assessments and structural neuroimaging by training machine learning tools in a high-specificity regime.

**Results:**

A multimodal signature of Alzheimer's dementia was first extracted from the ADNI1 dataset. We then validated the predictive value of this signature on ADNI1 patients with mild cognitive impairment (N = 235). The signature was optimized to predict progression to dementia over 3 years with low sensitivity (55.1%) but high specificity (95.6%), resulting in only moderate accuracy (69.3%) but high positive predictive value (80.4%, adjusted for a “typical” 33% prevalence rate of true progressors). These results were replicated in ADNI2 (N = 235), with 87.8% adjusted positive predictive value (96.7% specificity, 47.3% sensitivity, 85.1% accuracy).

**Conclusions:**

We found that cognitive measures alone could identify high-risk individuals, with structural measurements providing a slight improvement. The signature had comparable receiver operating characteristics to standard machine learning tools, yet a marked improvement in positive predictive value was achieved over the literature by selecting a high-specificity operating point. The multimodal signature can be readily applied for the enrichment of clinical trials.

## Introduction

Alzheimer's disease (AD), a leading cause of dementia, is marked by the abnormal accumulation of amyloid β (Aβ) and hyperphosphorylated τ proteins in the brain, which leads to widespread neurodegeneration. AD has a long prodromal phase, and it has been difficult to predict which individuals will decline and experience AD dementia. While mild cognitive impairment (MCI) puts individuals at risk, only a fraction (33.6% on average) of patients with MCI will develop dementia within a period of 3 years or more, as shown in a meta-analysis of 41 studies [1]. Identifying patients with MCI who will progress to AD dementia with enough specificity has thus been a challenge for clinical trials [2]. This lack of prognostic power may be due to individual variability. Different clinical phenotypes have been described in which patients will exhibit distinct cognitive deficits [3]. Previous work has also characterized neuropathological subtypes based on the distribution of neurofibrillary tangles [4], which correspond well to distinct patterns of brain atrophy [5]. Different subtypes of brain atrophy have also been associated with different rates of progression to dementia [6]. The implications for prognosis are profound: only a subgroup of patients will experience a sharp cognitive decline that can be reliably predicted. We therefore propose to identify a subset of individuals with a homogenous signature of brain atrophy and cognitive deficits who will progress to AD dementia with high precision.

There is a large field focused on using machine learning to automatically detect patients with MCI who will progress to AD dementia on the basis of imaging and cognitive features. For models combining structural magnetic resonance imaging (MRI) and cognition, state-of-the-art performance is 79% accuracy (76% specificity, 83% sensitivity) [7]. Some groups have achieved higher accuracies ranging from 82 to 97% when using other imaging methods, such as Aβ positron emission tomography [8] or resting-state functional MRI [9]. Although this increase in accuracy may suggest that Aβ imaging and resting-state functional MRI are better features, these imaging measures are more invasive, costly, and currently lack the large scale of validation of tools that are already widely used in clinical settings, such as cognitive assessments and structural MRI. Given the need to develop tools that will easily scale up in clinical settings, we propose to focus on predictive models that use structural imaging and cognition as features.

Models are typically trained to maximize accuracy, defined as the proportion of participants who were correctly identified, either as progressors or non-progressors. For enrichment in clinical trials, a more relevant metric is positive predictive value (PPV), which is the proportion of participants who actually progress to dementia when they have been identified as such by the model. The PPV of a model is dependent on the baseline rate of progression in the sample, with a typical rate (within 3 years or more) in patients with MCI being 33.6% [1]. Assuming a 33.6% baseline rate, it is possible to calculate the PPVs of published models in the literature, based on reported sensitivity and specificity scores. The adjusted PPV for models using cognitive and structural measures ranged from 50 to 75% [7, 8, 10–16]. In other words, up to half of subjects who were identified as progressors by published algorithms would not actually progress to dementia in a typical MCI sample. We therefore aimed to adapt the training regimen of predictive models to favor specificity over sensitivity, with the hypothesis that in this regime the models will identify progressors with high PPV. We expected that optimizing for high specificity will result in a low number of false-positive results, which is particularly important when the prevalence of progressors is low and therefore the susceptibility of the predictive model to identify false-positive progressors is high.

The overall goal of this work was to develop a model to identify individuals who are at high risk of progression to AD dementia with high PPV and specificity, using structural MRI and cognitive features. We aimed to show that by training standard machine learning tools in a high-specificity regime, we can identify the most robust progressor MCI patients with high confidence. We further wanted to assess whether those high-risk individuals had prodromal AD, by examining longitudinal cognitive decline, as well as Aβ and τ burden in these individuals. We finally aimed to evaluate the complementarity of features derived from cognition and atrophy patterns by examining the overlap of high-risk individuals who were identified as such by each modality. Although the complementarity of cognitive and structural measures has been extensively studied for prognosis of dementia in a general MCI population, the main contribution of this work is to examine their complementarity in the specific context of a high-risk signature that achieves high specificity and PPV at the cost of low sensitivity when the class of interest is relatively rare. Specific aims, as well as a summary of experiments and the main results, are listed in Table 1.

**Table 1:** Summary of objectives, experiments, and main findings

Specific objectives	Experiments	Main findings
(i) Identify subtypes of brain atrophy patterns	We used unsupervised clustering on atrophy maps generated from structural images in patients with AD and cognitively normal participants	Seven distinct patterns of atrophy were identified, some of which were strongly associated with a diagnosis of AD (Fig. 1b)
(iia) Replicate previous findings from works that used cognitive and structural features to predict progression to AD from MCI	A linear SVM that was optimized for accuracy was trained on the following features: (i) structural atrophy patterns, (ii) multi-domain cognitive assessments, and (iii) a combination of both	The SVM based on cognitive features had higher predictive value than the structural MRI signature, similar to previous findings [7] (see Figs 2 and 3)
(iib) Train a model in a high-specificity regime to identify high-confidence AD participants with a high-risk signature	We used a 2-stage algorithm to ensure that we were maximizing specificity over sensitivity. We trained on the following features: (i) structural atrophy patterns, (ii) multi-domain cognitive assessments, and (iii) a combination of both	The 2-stage algorithm resulted in a model that achieved high specificity and high PPV, with reduced sensitivity (Fig. 2). Three high-risk signatures were generated (Fig. 5)
(iii) Assess whether the high-risk signature generated by the 2-stage algorithm can identify progressors in participants with MCI within a 3-year period	We measured PPV, specificity, sensitivity, and accuracy of the model in predicting progressors in 2 separate MCI cohorts	The model achieved high specificity and high PPV, again at the cost of sensitivity and accuracy (Figs 2 and 4)
(iv) Test the performance of the 2-stage algorithm against standard algorithms	We compared the ROC performance of the 2-stage algorithm against standard algorithms (e.g., KNN, GNB, SVM with an RBF kernel)	The performance of the 2-stage algorithm did not differ from standard algorithms, in terms of area under an ROC curve, but was the only one to operate in a high-specificity, low-sensitivity regime (Fig. 3)
(v) Validate whether this high-risk signature represents a prodromal phase of AD	We compared cognitive decline, Aβ, and τ burden in tagged high-risk individuals against those who were not	Tagged high-risk individuals experienced sharper cognitive decline and higher levels of Aβ and τ than non-tagged individuals (Fig. 4)
(vi) Assess the complementarity of cognitive and structural measures	We examined whether there was overlap in the participants who were identified by the 3 high-risk signatures	The majority of participants who were identified by the multimodal high-risk signature had been identified as such by the unimodal cognitive and unimodal structural signatures. The unimodal cognitive signature identified the majority of all high-risk participants (Fig. 6)

## Materials and Methods

### Data

Data used in the preparation of this article were obtained from the Alzheimer's Disease Neuroimaging Initiative (ADNI) database [17]. The ADNI was launched in 2003 as a public-private partnership, led by principal investigator Michael W. Weiner, MD. The primary goal of ADNI has been to test whether serial MRI, positron emission tomography (PET), other biological markers, and clinical and neuropsychological assessment can be combined to measure the progression of MCI and early AD. For up-to-date information, see [18].

We took baseline T1-weighted MRI scans from the ADNI1 (228 cognitively normal [CN] participants, 397 with MCI, 192 with AD) and ADNI2 (218 CN, 354 MCI, 103 AD) studies. For a detailed description of MRI acquisition details, see [19]. All participants gave informed consent to participate in these studies, which were approved by the research ethics committees of the institutions involved in data acquisition. Consent was obtained for data sharing and secondary analysis, the latter being approved by the ethics committee at the Centre de Recherche de l'Institut Universitaire de Gériatrie de Montréal. For the MCI groups, each individual must have had ≥36 months of follow-up for inclusion in our analysis. We also further stratified the MCI groups into stable (sMCI), who never received any change in their diagnosis, and progressors (pMCI), who received a diagnosis of AD dementia within 36 months of follow-up. pMCI who progressed to AD dementia after 36 months were excluded. After applying these inclusion and exclusion criteria, we were left with 280 and 268 eligible participants with MCI in ADNI1 and ADNI2, respectively.

### Structural features from voxel-based morphometry

Images were processed with the NeuroImaging Analysis Kit (NIAK), version 0.18.1 [20], the MINC toolkit [21], version 0.3.18, and SPM12 [22] under CentOS with Octave [23], version 4.0.2. Preprocessing of MRI data was executed in parallel on the Cedar supercomputer [24], using the Pipeline System for Octave and Matlab (PSOM) [25]. Each T1 image was linearly co-registered to the Montreal Neurological Institute (MNI) ICBM152 stereotaxic symmetric template [26], using the CIVET pipeline [27], and then re-oriented to the AC-PC line. Each image was segmented into grey matter, white matter, and cerebrospinal fluid (CSF) probabilistic maps. The DARTEL toolbox [28] was used to normalize the grey matter segmentations to a predefined grey matter template in MNI152 space. Each map was modulated to preserve the total amount of signal and smoothed with an 8-mm isotropic Gaussian blurring kernel. After quality control of the normalized grey matter segmentations, we were left with 621 participants in ADNI1 (621 of 700 [88.7% success rate]) and 515 participants in ADNI2 (515 of 589 [87.4% success rate]).

**Figure 1:**
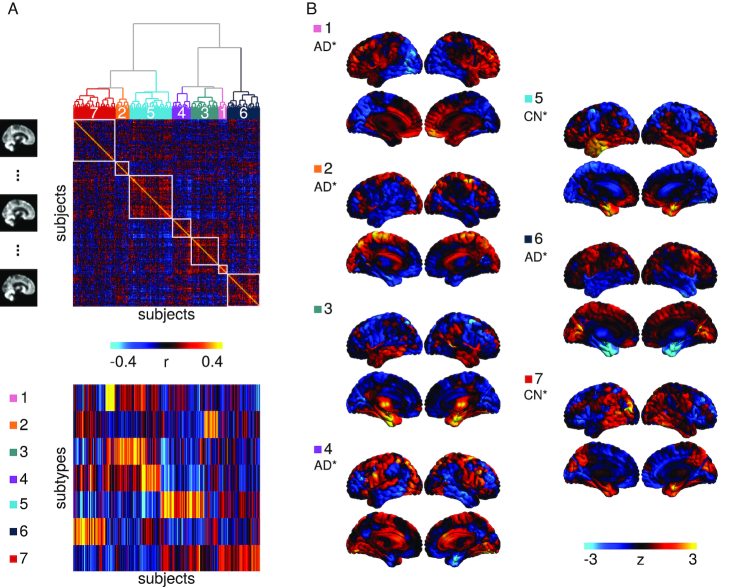
Subtyping procedure and resulting subtypes. (**a**) A hierarchical clustering procedure identified 7 subtypes, or subgroups, of individuals with similar patterns of grey matter topography within the ADNI1 cohort of CN and AD participants (top). A measure of spatial similarity, called subtype weight, between a single individual's GMV map and the average of a given subtype was calculated for all individuals and all subtypes (bottom). (**b**) Maps of the 7 subtypes showing the distribution of grey matter across all voxels relative to the average. CN* and AD* denote significant associations between the subtype weights and diagnoses of CN or AD, respectively.

We extracted subtypes to characterize variability of grey matter distribution with the CN and AD samples from ADNI1. To reduce the impact of factors of no interest that may have influenced the clustering procedure, we regressed out age, sex, mean grey matter volume (GMV), and total intracranial volume (TIV), using a mass univariate linear regression model at each voxel. We then derived a spatial Pearson's correlation coefficient between all pairs of individual maps after confound regression. This defined a participant × participant (377 × 377) similarity matrix, which was entered into a Ward hierarchical clustering procedure (Fig. 1a). By means of visual inspection of the similarity matrix, we identified 7 subgroups (Fig. 1b). Each subtype was defined as the average map of each subgroup. For each participant, we computed spatial correlations between their map and each subtype, which we call weights (Fig. 1a). The weights formed an *n* participant × *m* subtypes (*n* = 377, *m* = 7) matrix, which was included in the feature space for all predictive models including voxel-based morphometry (VBM) throughout this work. As in our previous works [29, 30], we chose to use weights, which can be interpreted as continuous measures for subtype affinity, over discrete subtype membership because the latter is less informative as most individuals express similarity to multiple subtypes [31]. Note that although we chose to present our findings with 7 subtypes, we examined how the number of subtypes may affect our subsequent predictions. There was no significant difference in model performance when we changed the number of subtypes (see Table S1 in supplementary material).

### Cognitive features

We took baseline neuropsychological scores for each participant from several cognitive domains: memory from the composite score ADNI-MEM [32], executive function from the composite score ADNI-EF [33], language from the Boston Naming Test (BNT), visuospatial from the clock drawing test, and global cognition from the Alzheimer's Disease Assessment Scale−Cognitive (ADAS13). We chose measures that span multiple cognitive domains because it has been suggested that the use of a combination of neuropsychological measures is likely the best approach to predicting incipient dementia [34]. These scores were included as features for the predictive models involving cognition. Thirteen participants across both ADNI1 and ADNI2 (8 AD, 5 MCI) had to be excluded as a result of missing values in their cognitive assessments. See Table 2 for demographic information of participants who were included in analyses.

**Table 2:** Demographic information for post−quality control participants in ADNI1 and ADNI2

Parameter	CN	sMCI	pMCI	AD
ADNI1				
No.	205	88	147	165
Age, y, mean ± SD	76.1 ± 5.0	74.0 ± 7.6	74.3 ± 7.1	75.4 ± 7.5
Female sex, %	51.7	40.9	40.8	51.5
APOE4+, %	27.8	37.5	68.7	65.4
ADAS13 score, mean ± SD	9.5 ± 4.3	14.3 ± 5.5	21.3 ± 5.3	28.6 ± 7.1
MMSE score, mean ± SD	29.1 ± 1.0	27.7 ± 1.7	26.7 ± 1.7	23.4 ± 2.0
ADNI2				
No.	188	180	55	89
Age, y, mean ± SD	72.8 ± 6.1	70.8 ± 7.3	72.1 ± 7.1	74.4 ± 7.8
Female sex, %	54.0	47.8	49.1	46.1
APOE4+, %	29.4	35.6	65.4	71.3
ADAS13 score, mean ± SD	9.1 ± 4.2	11.8 ± 5.3	21.4 ± 6.5	31.6 ± 8.7
MMSE score, mean ± SD	29.1 ± 1.1	28.4 ± 1.6	27.3 ± 1.9	23.1 ± 2.3

ADAS13: Alzheimer's Disease Assessment Scale—Cognitive subscale (13 items); MMSE: Mini Mental State Examination; SD: standard deviation.

### Prediction of high-confidence AD dementia cases in ADNI1

We trained a linear support vector machine (SVM) model with a linear kernel, as implemented by Scikit-learn [35], version 0.18, to classify AD vs CN from ADNI1 to get a baseline prediction accuracy. We then used a 2-step method to select an operating point for the linear SVM to obtain a highly precise and specific classification [29]. This was done by replicating the SVM prediction via subsampling and identifying the patients with highly robust prediction outcomes, i.e., who are consistently identified as true AD cases (true-positive results) during testing, regardless of the training subsample. This approach was found, in practice, to lead to a highly specific prediction, in addition to offering a guarantee of robustness; see [29] for more information. Specifically here, a 10-fold cross-validation loop was used to estimate the performance of the trained model. Classes were balanced inversely proportional to class frequencies in the input data for the training. A nested cross-validation loop (stratified shuffle split with 50 splits and 20% test size, i.e., a random permutation cross-validator was used to split the data into 50 training and test sets, where the size of the test set was always 20% of the original sample size) was used for the grid search of the SVM hyperparameter *C* (grid was 10^−2^ to 10^1^ with 15 equal steps). We randomly selected subsamples of the dataset, retaining a set percentage of participants in each subsample. For each subsample, a separate SVM model was trained to predict AD or CN in ADNI1. The SVM training was replicated a number of times. Both the subsample size and the number of subsamples were selected to maximize the PPV of the prediction of sMCI vs pMCI in ADNI1, as described below. Predictions were made on the remaining participants who were not used for training, and, for each participant, we calculated a hit probability defined as the frequency of correct classification across all SVM replications in which the test set contained that participant. High-confidence AD cases were defined as individuals with 100% hit probabilities with the AD label. Next, we trained a logistic regression classifier [36], with L1 regularization on the coefficients, to predict the high-confidence AD cases. A stratified shuffle split (500 splits, 50% test size) was used to estimate the performance of the model for the grid search of the hyperparameter *C* (grid was 10^−2^ to 10^1^ with 15 equal steps) on the overall ADNI1 sample, and the same hyperparameters were used for all SVM replications.

We used the entire CN and AD sample from ADNI1 to obtain 3 highly predictive signatures (HPS) (i.e., models), (i) one using VBM subtype weights as features (VBM only), (ii) one using only cognitive features (COG only), (iii) and one using the combination of VBM subtype weights and cognitive features (VCOG). In all 3 signatures, age, sex, mean GMV, and TIV were also included as features.

### Prediction of progression to AD dementia from the MCI stage in ADNI1

The logistic regression trained on AD vs CN was used to identify patients with MCI who have an HPS of AD dementia in ADNI1. Our hyperparameters for this logistic regression were optimized on the basis of the number of subsamples and subsample size that produced the maximum specificity and PPV for the classification of sMCI (n = 89) vs pMCI (n = 155) in ADNI1, while maintaining a minimum of 30% sensitivity. We varied the number of subsamples (100, 500, 1,000) and subsample size (10%, 20%, 30%, 50%) to perturb the model and identify participants who had robust outcomes during the testing phase regardless of the training subsample. We then re-trained our models to classify AD vs CN in ADNI1 with these optimized hyperparameters. This was done for all 3 signatures. In brief, we used the AD and CN sample from ADNI1 as a training set and the participants with MCI from ADNI1 as a validation set. The ADNI2 sample was then used as an independent replication (test) set to establish the performance of the 2-stage model without further changes to the hyperparameters.

### Statistical test of differences in model performance

We used Monte Carlo simulations to generate confidence intervals on the performance (i.e., accuracy, PPV, specificity, and sensitivity) of both linear SVM and HPS models for their predictions of AD vs CN and pMCI vs sMCI. Taking the observed sensitivity and specificity and using sample sizes similar to those in our experiment, we replicated the number of true- and false-positive detections 100,000 times using independent Bernoulli variables, and derived replications of accuracy, PPV, specificity, and sensitivity. By comparing these replications with the accuracy, sensitivity, specificity, and PPV observed in both models, we estimated a *P*-value for differences in model performance [37]. A *P*-value <0.05 was interpreted as evidence of a significant difference in performance, and <0.001, as strong evidence. We also used this approach to compare the performance of the combined features (VCOG) to the models containing VBM features (VBM) or COG only. Note that, based on our hypotheses regarding the behaviour of the HPS model, the tests were 1 sided for increased accuracy, specificity, and PPV and 1 sided for decreased sensitivity.

To assess the performance of the HPS models against standard machine learning algorithms, we used 4 algorithms (SVM with a radial basis function [RBF] kernel, K nearest neighbours, random forest, and Gaussian naive Bayes) to train models to classify AD vs CN in the ADNI1 dataset. We then tested and validated these models on classifying AD vs CN in ADNI2 and pMCI vs sMCI in both ADNI1 and ADNI2 separately. See the supplementary material for details of the implementations of these latter algorithms. We then generated receiver operating characteristic (ROC) curves and calculated the area under the curve (AUC) for each model and classification (AD vs CN; pMCI vs sMCI) in both ADNI1 and ADNI2.

### Statistical tests of association of progression, AD biomarkers, and risk factors in high-confidence MCI participants

On the basis of the classifications resulting from the linear SVM and HPS models, we separated the participants with MCI into 3 groups: (i) high confidence, participants who were selected by the HPS model as hits; (ii) low confidence, participants who were selected by the linear SVM model as hits but were not selected by the HPS model; and (iii) negative, participants who were not selected as hits by either algorithm.

To validate whether the high-confidence patients represented individuals who were in a prodromal phase of AD, we tested whether this subgroup was enriched for progression to dementia, apolipoprotein E ε4 (APOE4) carriers, females, and participants who were positive for Aβ and τ pathology. Positivity of AD pathology was determined by CSF measurements of Aβ 1–42 peptide and total τ with cut-off values of <192 and >93 pg/mL, respectively [38]. We implemented Monte Carlo simulations, where we selected 100,000 random subgroups out of the original MCI sample. By comparing the proportion of progressors, APOE4 carriers, females, Aβ-positive, and τ-positive participants in these null replications with the actual observed values in the HPS subgroup, we estimated a *P*-value [37] (1 sided for increase). A *P*-value <0.05 was interpreted as evidence of a significant enrichment, and <0.001, as strong evidence.

One-way analyses of variance were used to evaluate differences between the HPS groupings with respect to age. Post hoc Tukey's HSD tests were performed to assess pairwise differences amongst the 3 classes (high confidence, low confidence, negative). These tests were implemented in Python with the SciPy library [39], version 0.19.1, and StatsModels library [40], version 0.8.0.

To explore the effect of HPS grouping on cognitive trajectories, linear mixed-effects models were performed to evaluate the main effects of and interactions between the HPS groups and time on ADAS13 scores up to 36 months of follow-up. The models were first fit with a random effect of participant and then were fit with random slopes (time | participant) if analyses of variance comparing the likelihood ratio suggested a significant improvement in model fit. All tests were performed separately on the ADNI1 and ADNI2 datasets. These tests were implemented in R, version 3.3.2, with the library nlme, version 3.1.128 [41].

### Public code, data availability, and reproducibility

The code used in this experiment is available in a GitHub repository [42] and Zenodo [43].

We shared a notebook replicating all the machine learning experiments, starting after the generation of VBM subtypes. However, to protect the privacy of the study participants, we could not share individual subtype weights alongside other behavioural data and diagnostic information. We thus created parametric (Gaussian) bootstrap simulations, based on group statistics alone, that will allow interested readers to replicate results similar to those presented in this article, using the exact same code and computational environment that were used on real data, but with purely synthetic data instead. The notebook can be executed online via the Binder platform [44], and runs into a Docker container [45], built from a configuration file that is available on GitHub [46]. The container itself is available on Docker Hub [47]. The simulated data were archived on Figshare [48].

The simulation included the following 16 variables: age, sex, mean GMV, TIV, 5 cognitive assessment scores, and 7 VBM subtype weights from both ADNI1 and ADNI2. Participants who had missing values for these variables were discarded from the simulation, leaving N = 1,115 participants. We stratified the population into 12 subgroups: the 4 clinical labels (AD, pMCI, sMCI, CN), each further subdivided by the 3 prediction subclasses identified in this article (negative, low confidence, high confidence). For each subgroup, we estimated the average and covariance matrices between the 16 variables of interest. We then generated a number of multivariate normal data points that matched the number of participants found in each subgroup, using the empirical mean and covariance matrix of each subgroup. Finally, the range of the simulated data was clipped to the range of the original real data, and the simulated sex data points were binarized by nearest neighbour.

The statistics from the predictive model in the original implementation are similar to those of the simulated data. The model predicted the progression of dementia from MCI in ADNI1 with a PPV of 93.1% (specificity of 93.2%) on real data. This coincides with a 93.3% PPV (specificity of 94.3%) that we get when using the simulated data. Similarly, with the ADNI2 dataset the model achieved a 81.3% PPV (specificity of 96.7%) from the real data and a 75.7% PPV (specificity of 95.0%) from the simulated data.

## Results

### Subtypes of brain atrophy

Subtype 1 was characterized by reduced relative GMV in the occipital, parietal, and posterior temporal lobes. Subtype 2 displayed reduced relative GMV across the cortex, except for the medial parts of the parietal and occipital lobes and the cingulate. Subtype 3 had increased relative GMV in the medial and lateral temporal lobes, insula, and striatum. Subtype 4 had decreased relative GMV in the temporal lobes, inferior parietal lobes, posterior cingulate, and the prefrontal cortices. Subtype 5 was characterized by greater relative GMV in the temporal lobes, while Subtype 6 had the opposite pattern of reduced relative GMV in the temporal lobes. Subtype 7 displayed greater relative GMV in the parietal lobes, posterior lateral temporal lobes, medial temporal lobes, and medial occipital lobes. See Fig. 1b for surface representations of the subtypes. Diagnosis (CN, sMCI, pMCI, AD) accounted for a substantial amount of variance in subtype weights for Subtypes 1 (*F* = 8.51, *P* = 1.30 × 10^−5^), 2 (*F* = 10.32, *P* = 1.00 × 10^−6^), 4 (*F* = 14.53, *P* = 2.60 × 10^−9^), 5 (*F* = 13.86, *P* = 6.77 × 10^−9^), 6 (*F* = 34.27, *P* = 2.57 × 10^−21^), and 7 (*F* = 37.02, *P* = 5.85 × 10^−23^). Post hoc *t*-tests showed that participants with AD had significantly higher weights compared to CN participants (Fig. 1b) for Subtypes 1 (*t* = 2.88, *P* = 0.02), 2 (*t* = 4.05, *P* = 3.0 × 10^−4^), 4 (*t* = 4.83, *P* < 1.0 × 10^−4^), and 6 (*t* = 7.86, *P* = < 1.0 10^−4^), making these subtypes associated with a diagnosis of AD. CN participants had significantly higher weights compared to AD for Subtypes 5 (*t* = −4.86, *P* < 1.0 × 10^−4^) and 7 (*t* = −6.95, *P* < 1.0 × 10^−4^), making these subtypes associated with a CN status.

### Prediction of AD dementia vs CN individuals

**Figure 2:**
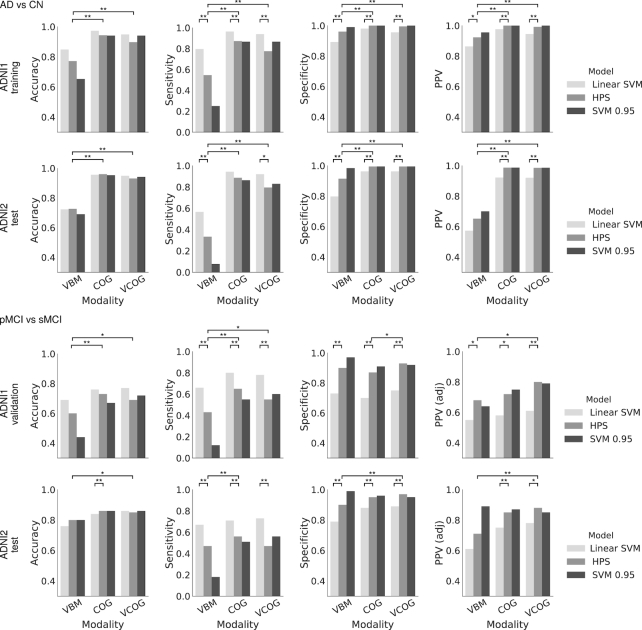
Accuracy, specificity, sensitivity, and PPV for different classifiers: linear SVM, HPS, and the linear SVM thresholded at 0.95 (SVM 0.95), for the classifications of patients with AD and CN individuals and patients with MCI who progress to AD (pMCI) and stable MCI (sMCI) in ADNI1 and ADNI2. VBM represents the model trained with VBM subtypes, COG represents the model trained with baseline cognitive scores, and VCOG represents the model trained with both VBM subtypes and cognition. PPV was adjusted [PPV (adj)] for a prevalence of 33.6% pMCI in a sample of MCI participants for both ADNI1 and ADNI2 MCI cohorts. Significant differences are denoted by * for *P* < 0.05 and ** for *P* < 0.001.

**Figure 3:**
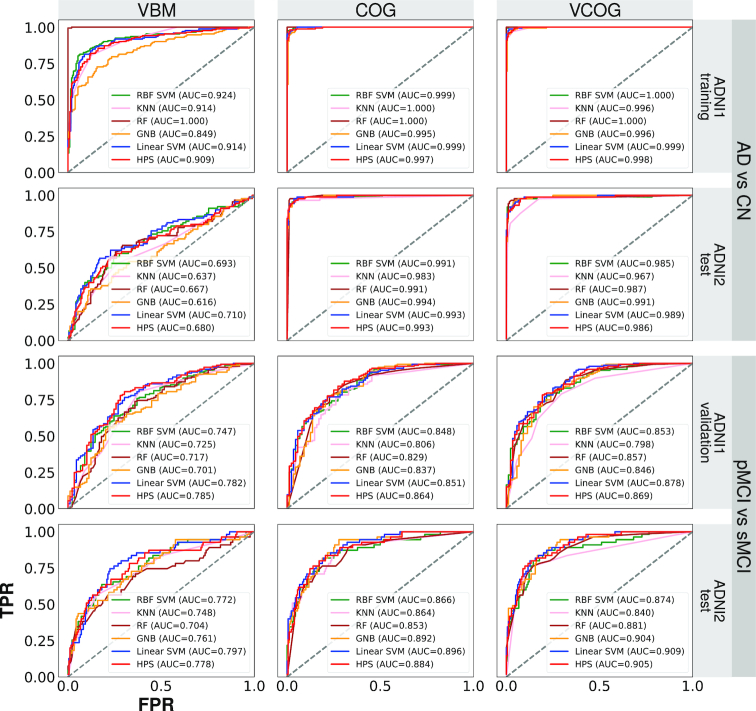
ROC curves for various machine learning algorithms with different features (VBM for VBM subtypes only, COG for cognitive features only, VCOG for a combination of VBM subtypes and cognitive features). Algorithms included an SVM with a radial basis function kernel (RBF SVM), K nearest neighbours (KNN), random forest (RF), Gaussian naive Bayes (GNB), an SVM with a linear kernel representing the first stage (Linear SVM) of the 2-stage predictive model, and the 2-stage HPS. TPR: true-positive rate; FPR: false-positive rate; AUC: area under the curve.

The linear SVM model trained using the VCOG features achieved 94.5% PPV (95.6% specificity, 93.9% sensitivity, 94.9% accuracy) when classifying AD vs CN in ADNI1. Such high performance was expected given the marked cognitive deficits associated with clinical dementia. The use of COG features only reached excellent performance as well (97.6% PPV, 98.0% specificity, 96.4% sensitivity, 97.3% accuracy), while using VBM features only yielded markedly lower performances (86.4% PPV, 89.3% specificity, 79.6% sensitivity, 84.8% accuracy) (see Fig. 2 and ROC analysis in Fig. 3). Note that the performance metrics in ADNI1 were estimated through cross-validation, and represent an average performance for several models trained on different subsets of ADNI1. We then trained a model on all of ADNI1 and estimated its performance on an independent dataset, ADNI2. Using VCOG predictors, the ADNI1 model reached 92.0% PPV (96.3% specificity, 92.0% sensitivity, 94.5% accuracy) when applied on ADNI2 AD vs CN data. Again the performance was comparable with COG predictors only (92.2% PPV, 96.3% specificity, 94.3% sensitivity, 95.6% accuracy), and VBM features only achieved lower performance (57.3% PPV, 79.8% specificity, 56.7% sensitivity, 72.3% accuracy) (see Fig. 2 and ROC analysis in Fig. 3). Note that PPV is dependent on the proportion of patients and controls for a given sensitivity and specificity. Because the ADNI2 sample had a substantially smaller proportion of AD participants compared to ADNI1, the resulting PPV was reduced. When we adjusted the baseline rate of AD participants in ADNI2 to the same rate as in ADNI1, the PPVs were 95.2%, 95.3%, and 70.2% for the VCOG, COG, and VBM models, respectively.

### Identification of high-confidence AD cases for prediction

The VCOG HPS model achieved 99.2% PPV (99.5% specificity, 77.6% sensitivity, 89.7% accuracy) in classifying high-confidence AD participants in ADNI1. These performance scores were estimated by cross-validation of the entire 2-stage process (training of SVM, estimation of hit probability, identification of HPS). However, the hyperparameters of the 2-stage model were optimized on classifying pMCI vs sMCI in ADNI1, as described previously. We next trained a single model on all of ADNI1, which we applied on an independent sample (ADNI2). The ADNI1 AD VCOG HPS model reached 98.6% PPV (99.5% specificity, 79.5% sensitivity, 93.1% accuracy) on ADNI2. As was previously observed with the conventional SVM analysis, the VCOG HPS model had similar performance to the COG HPS model (ADNI1: 100% PPV, 100% specificity, 87.3% sensitivity, 94.2% accuracy; ADNI2: 98.7% PPV, 99.5% specificity, 88.6% sensitivity, 96.0% accuracy) and outperformed the VBM HPS model (ADNI1: 92.3% PPV, 96.1% specificity, 54.6% sensitivity, 77.2% accuracy; ADNI2: 65.2% PPV, 91.5% specificity, 33.3% sensitivity, 72.7% accuracy); see Fig. 2. When adjusted to the same baseline rate of AD participants as ADNI1, the PPVs in ADNI2 were 99.2%, 99.3%, and 76.7% for the VCOG, COG, and VBM HPS models, respectively.

### High-confidence prediction of progression to AD dementia

When the analysis was based on the full VCOG features, 87 patients with MCI were selected as high confidence in ADNI1, of which 81 (93.1% PPV) were pMCI within 36 months of follow-up. This represented a large, significant increase over the baseline rate of progressors in the entire ADNI1 MCI sample (37.4%) (*P* < 0.001). This was also a significant increase over the SVM's predictions, where 83.9% of participants whom it had labelled as hits were true progressors (*P* < 0.001). When adjusted to a 33.6% baseline rate of progressors, more typical of MCI populations, the PPV of high-confidence patients for progression to dementia was 80.4% (93.2% specificity, 55.1% sensitivity, 69.3% accuracy).

We replicated these analyses in the MCI sample from ADNI2 (N = 235). Using VCOG features, 32 participants were identified as high confidence, 26 of whom progressed to AD dementia within 36 months' follow-up (81.2% PPV, specificity of 96.7%, sensitivity of 47.3%, 85.1% accuracy, 87.8% PPV adjusted to a 33.6% baseline rate). This represented a significantly higher prevalence than the 30.6% baseline rate in the entire ADNI2 MCI cohort (*P* < 0.001). This was also a significant increase over the SVM's predictions, where 67.8% of participants whom it had labelled as hits were true progressors (*P* < 0.001).

As in the classifications of AD vs CN, the VCOG HPS model tended to have higher performance compared to the VBM HPS (ADNI1: 89.9% specificity, 42.9% sensitivity, 60.5% accuracy, 87.7% PPV, 68.2% adjusted PPV; ADNI2: 90.1% specificity, 47.3% sensitivity, 80.2% accuracy, 59.1% PPV, 70.7% adjusted PPV) in classifying pMCI vs sMCI. The VCOG HPS also had similar performance compared to the COG HPS (ADNI1: 87.5% specificity, 64.6% sensitivity, 73.2% accuracy, 89.6% PPV, 72.3% adjusted PPV; ADNI2: 95.0% specificity, 56.4% sensitivity, 86.0% accuracy, 77.5% PPV, 85.1% adjusted PPV) for distinguishing between pMCI and sMCI. Notably, the analysis based on the VCOG features led to higher PPV than VBM and COG features taken independently, both in ADNI1 and ADNI2. That increase was large and significant between VCOG and VBM (up to 17%) and marginal and non-significant between VCOG and COG (up to 8%); see Fig.   2.

### Trade-off between sensitivity and specificity of different algorithms

The HPS models consistently outperformed the linear SVM classifiers with respect to specificity (*P* < 0.001) in the classifications of AD vs CN and pMCI vs sMCI in both ADNI1 and ADNI2, regardless of the features that the models contained. The HPS also had greater PPV (*P* < 0.05) adjusted for a typical prevalence of 33.6% pMCI in a given sample of MCI participants [1]. However, these increases in specificity and PPV for the HPS model came at a significant cost of reduced sensitivity compared to the linear SVM classifier, across all models in both ADNI1 and ADNI2 (*P* < 0.05) (Fig. 2). Note that this shift towards lower sensitivity and higher specificity and PPV could be achieved by adjusting the threshold of the SVM analysis (see Fig. 2 and ROC analysis in Fig. 3) and is not unique to the 2-stage procedure we implemented. This trade-off between sensitivity and specificity is universal across machine learning algorithms, and similar results can be achieved by adjusting the prediction threshold of different strategies. As shown by the ROC curves and AUC values in Fig. 3, other machine learning algorithms (SVM with a radial basis function kernel, K nearest neighbours, random forest, and Gaussian naive Bayes) also performed similarly to the HPS. Thus, the value of the HPS is in the selection of a threshold point in order to operate in a high-specificity regime.

### Characteristics of MCI participants with a highly predictive VCOG signature of AD

**Figure 4:**
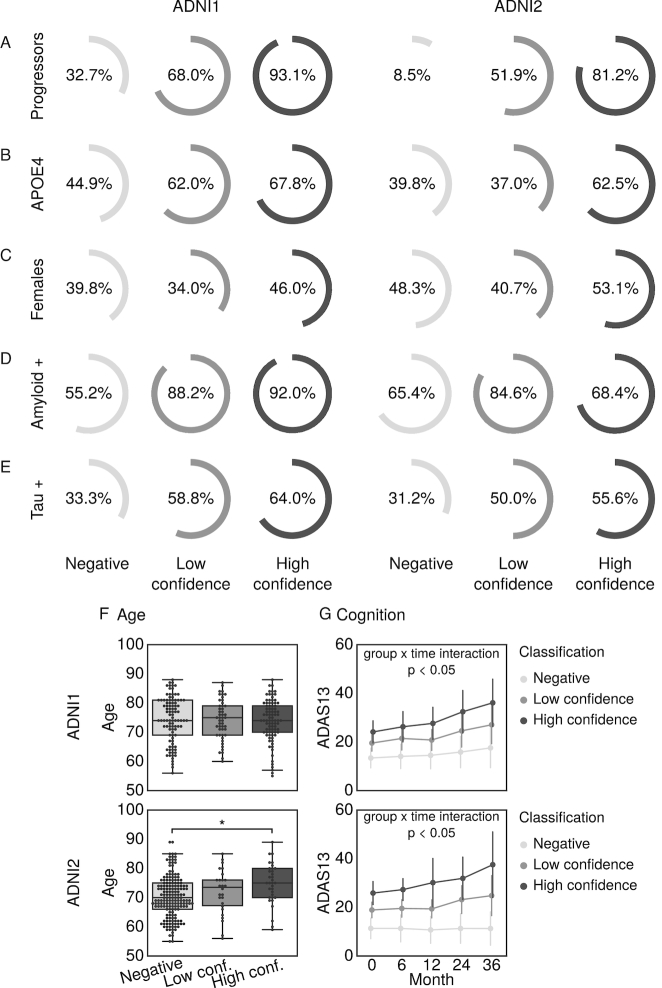
Characteristics of participants with MCI with the VCOG signature in ADNI1 and ADNI2. We show the percentage of participants with MCI who (a) progressed to dementia and were (b) APOE4 carriers, (c) female, (d) positive for Aβ measured by a cut-off of 192 pg/mL in the CSF [38], and (e) positive for τ measured by a cut-off of 93 pg/mL in the CSF [38] in each classification (high confidence, low confidence, and negative). (f) Age and (g) cognitive trajectories, measured by the Alzheimer's Disease Assessment Scale-Cognitive subscale with 13 items (ADAS13), across the 3 classes. Significant differences are denoted by asterisks for family-wise error rate-corrected *P* < 0.05.

High-confidence MCI participants with the VCOG signature were more likely to be progressors (Fig. 4a) compared to low-confidence patients and negative patients (ADNI1: *P* < 0.001; ADNI2: *P* < 0.001). High-confidence MCI participants were also more likely to be APOE4 carriers (Fig. 4b) (ADNI1: *P* < 0.005; ADNI2: *P* < 0.05). There was no difference in sex across the HPS groupings in the participants with MCI of either the ADNI1 or ADNI2 cohort (Fig. 4c). This was consistent with the whole sample, where there were equal proportions of progressors across both sexes in each dataset (ADNI1: χ^2^ = 0.015, *P* = 0.90; ADNI2: χ^2^ = 0.0002, *P* = 0.99). The high-confidence class was also significantly enriched for Aβ-positive participants in ADNI1 (*P* < 0.05). However, this result was not replicated in the ADNI2 participants with MCI (Fig. 4d). Similarly with τ, we found a significant increase in τ-positive participants in the high-confidence group of ADNI1 (*P* < 0.05) but not in ADNI2 (Fig. 4e). We found a significant age difference across the HPS classes in ADNI2 (*F* = 5.68, *P* < 0.005), where the high-confidence patients were older than the negative participants by a mean of 4.4 years. However, age did not differ across the HPS classes in ADNI1 (Fig. 4f). Finally, high-confidence patients had significantly steeper cognitive declines compared to the low-confidence and negative groups (Fig. 4g): there were significant interactions between the HPS groupings and time in ADNI1: (high confidence β = −0.147, *t* = −7.56, *P* < 0.001; low confidence β = −0.055, *t* = −2.46, *P* < 0.05) and ADNI2 (high confidence β = −0.194, *t* = −8.69, *P* < 0.001; low confidence β = −0.072, *t* = −3.31, *P* = 0.001). The high-confidence patients in ADNI1 and ADNI2, respectively, gained 1.8 and 2.3 more points each year on the ADAS13 compared to the low-confidence and negative groups. Note that higher scores on the ADAS13 represent worse cognitive function.

### COG, VBM, and VCOG highly predictive signatures

**Figure 5:**
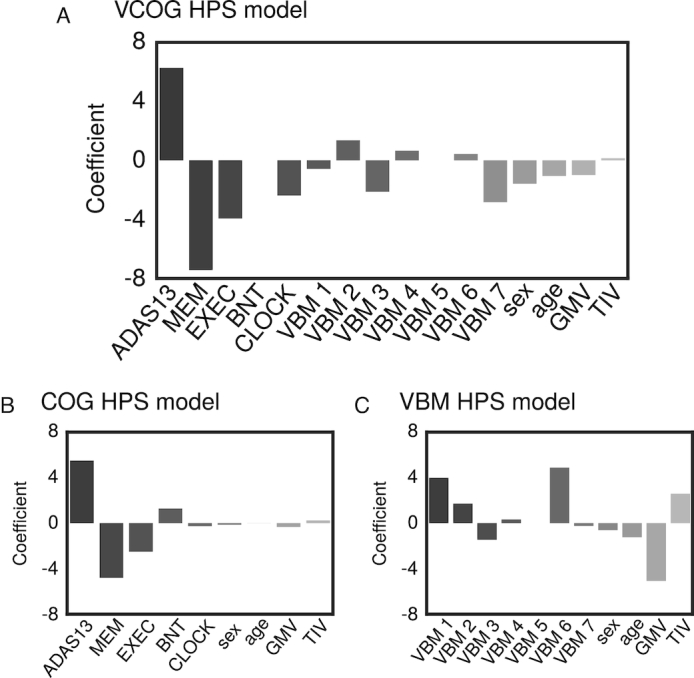
Coefficients of the high-confidence prediction (a) VCOG HPS model, (b) COG HPS model, (c) VBM HPS model. ADAS13 = Alzheimer's Disease Assessment Scale—Cognitive; MEM = ADNI-MEM score; EXEC = ADNI-EF score; BNT = Boston Naming Test; CLOCK = clock drawing test; VBM 1–7 = VBM subtype weights.

The COG signature was mainly driven by scores from the ADAS13, which measures overall cognition; ADNI-MEM, a composite score that measures memory [32]; and ADNI-EF, a composite score that measures executive function [33] (coefficients were 5.49, −4.80, and −2.50, respectively). In this model, sex, age, mean GMV, and TIV contributed very little relative to the cognitive features (Fig. 5b). Note that these coefficients should be interpreted as pseudo *z*-scores because the features had been normalized to zero mean and unit variance.

Almost all grey matter subtypes contributed to the VBM signature. Mean GMV, Subtype 1 (reduced relative GMV in the occipital, parietal, and posterior temporal lobes), and Subtype 6 (reduced relative GMV in the temporal lobes, notably the medial temporal regions) had the highest weights in the model (coefficients were −5.07, 4.87, and 3.98, respectively) (Fig. 5c). We had anticipated the larger contribution of these 2 subtypes because they have been described in previous AD subtyping work [5, 49–51].

The ADAS13, memory (ADNI-MEM), and executive function (ADNI-EF) scores contributed the most to the VCOG signature (coefficients were 6.27, −7.43, and −3.95, respectively; Fig. 5a). Of the VBM features, Subtypes 2, 3, and 7 contributed the most to the signature (coefficients were 1.36, −2.12, and −2.83, respectively). Subtypes 1 and 6, which had the highest positive weights in the VBM HPS model, were given marginal weights in the VCOG HPS model, which is potentially indicative of redundancy with COG features. Note that the weights for Subtypes 3 and 7 were negative in the model, which means that predicted AD and pMCI cases had brain atrophy patterns that were spatially dissimilar to those subtypes.

### Comparison of COG, VBM, and VCOG high-confidence patients

**Figure 6:**
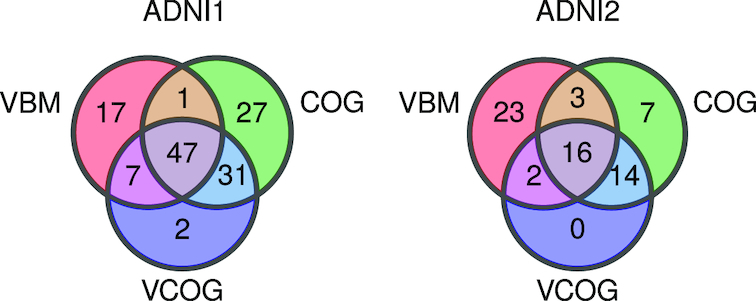
Venn diagram depicting the number of participants with MCI labelled as high confidence by the VBM, COG, and VCOG HPS models in ADNI1 and ADNI2.

We found substantial overlap of participants labelled as high confidence in the MCI cohorts across the VBM, COG, and VCOG signatures (Fig. 6). There were very few participants who were labelled as high confidence exclusively by the VCOG signature. As to be expected, the majority of participants labelled as high confidence by the VCOG signature (ADNI1: 97.7%; ADNI2: 100%) were also labelled as high confidence by either the VBM only or COG only signatures or both. Of the participants who were labelled as high confidence by the VBM only signature, 23.6% and 55.2% in ADNI1 and ADNI2, respectively, were identified exclusively by the VBM HPS. Relatively few participants (7 and 2 participants in ADNI1 and ADNI2, respectively) were captured by VBM and VCOG but missed by the COG HPS. The COG HPS actually identified the majority of all high-confidence patients across the 3 signatures (ADNI1: 106 of 132 total participants; ADNI2: 40 of 65 total participants). From Fig. 6, we can see that the VCOG HPS acts as a refinement of the COG signature, as the VCOG HPS captures a subset of participants who were labelled by the COG HPS.

Of the high-confidence participants labelled by all 3 signatures, 97.9% and 93.7% from ADNI1 and ADNI2, respectively, progressed to dementia (Supplementary Table S2). These participants had worse cognition based on the MMSE and higher proportions of APOE4 carriers, Aβ-positive, and τ-positive individuals, compared to the baseline rates in all participants with MCI. Of the high-confidence patients who were labelled only by the VBM model, 70.6% and 43.4% from ADNI1 and ADNI2, respectively, were progressors. This group of participants had fewer Aβ- and τ-positive individuals compared to the baseline rates. Of the high-confidence patients who were labelled only by the COG model, 70.4% and 57.1% from ADNI1 and ADNI2, respectively, progressed to dementia. This group appeared to have a greater proportion of Aβ-positive individuals compared to the baseline rates in both the ADNI1 and ADNI2 cohorts. The majority of these COG high-confidence participants were also male. Given the distinct characteristics amongst the exclusively COG, exclusively VBM, and VCOG high-confidence patients, these groups may represent subgroups with different risks for AD dementia. Because it appears that a greater proportion of pMCI is captured when cognitive and structural MRI features are combined, these findings may support joining multiple modalities together to achieve higher PPV. However, these results are qualitative and of an exploratory nature owing to low sample sizes.

## Discussion

We developed an MRI- and cognitive-based model to predict AD dementia with high PPV and specificity. Specifically, our 2-stage predictive model reached 93.2% specificity and 93.1% PPV (80.4% when adjusted for 33.6% prevalence of progressors) in ADNI1 when classifying progressor vs stable MCI patients (within 3 years' follow-up). We replicated these results in ADNI2, where the model reached 96.7% specificity and 81.2% PPV (87.8% adjusted PPV). With respect to specificity and PPV, these results are a substantial improvement over previous works combining structural MRI and cognition on the same prediction task, which have reported up to 76% specificity and 65% PPV (adjusted for 33.6% prevalence of progressors) [7]. Finally, our results also reproduced our past work, which developed a model that optimizes specificity and PPV [29]. However, it appears that a combination of structural and functional MRI measures may lead to an improved prediction because 2 studies have reported 90–100% PPV with these measures [9, 29], with the limitation of smaller sample sizes (56 total participants with MCI in [29], 86 total participants with MCI in [9]) due to the limited availability of functional MRI data in ADNI. Our proposed signature is based on widely available measures and can be readily tested in many clinical trials. Functional MRI measures, by contrast, are only gaining traction in large clinical studies and will at the minimum require more time to get widely adopted, if the very high PPVs are replicated in larger samples.

Recently, there has been great interest in using deep learning for automated image-guided diagnosis. Compared to traditional machine learning approaches, deep learning requires minimal to no image pre-processing and automatically discovers optimal representations of the data needed for classification without prior feature selection [52]. Studies that have used deep learning to predict progression to AD dementia in patients with MCI from structural MRI have reported 74.9–79.9% accuracy, 75.8–84% sensitivity, and 74.1–74.8% specificity [53, 54]. Studies of multimodal prediction of AD progression in patients with MCI with deep learning have reported higher accuracies, in the range of 81–86%, with various combinations of features, such as fluorodeoxyglucose PET and structural MRI [55], structural MRI and cognitive measures [56], structural MRI, CSF, and cognition [57], and fluorodeoxyglucose PET and Aβ PET [58]. Overall, these results represent a modest improvement over previous traditional machine learning techniques. While an accuracy of up to 86% for discriminating pMCI from sMCI may seem promising, the reported sensitivities and specificities of these multimodal deep learning studies ranged from 79% to 88% and 79% to 84%, respectively [55–58], which translates to PPVs ranging from 65.5% to 73.5% when assuming a 33.6% prevalence rate of progressors in a sample of patients with MCI. With respect to PPV and specificity, our results still represent a considerable improvement over the current literature in predicting progression to AD dementia in patients with MCI. That is not to say that the models used here are improvements over state-of-the-art prognosis models from a machine learning perspective. Rather, we pushed relatively standard techniques into a regime of high specificity and precision. This regime had not been explored much until this point and could prove useful in applications such as enrichment of clinical trials. If our high-precision 2-stage approach were to be applied to a deep learning model with a higher baseline accuracy, we would expect an even more precise prognosis.

An ideal model to predict conversion to AD dementia would have both high sensitivity and specificity. However, the pathophysiological heterogeneity of clinical diagnosis will prevent highly accurate prediction linking brain features to clinical trajectories. We argue that, faced with heterogeneity, it is necessary to sacrifice sensitivity to focus on a subgroup of individuals with similar brain abnormalities. Due to the expected trade-off between specificity and sensitivity, the high specificity of our 2-stage model indeed came at a cost of reduced sensitivity (55.1% in ADNI1 and 47.3% in ADNI2 for classifying pMCI vs sMCI), which is much lower than sensitivity values of 64–95% reported by other groups [7, 8, 10–16]. The 2-stage procedure did not offer gains compared to a simpler SVM model if the threshold of the SVM model could be selected *a priori* to match the specificity of the 2-stage procedure (see ROC curves in Fig. 3). The 2-stage prediction model offered the advantage of a principled approach to train the prediction model in order to maximize specificity, based on samples that are robust and easily classifiable, without testing a range of prediction thresholds. The choice of an L1 regularized logistic regression also led to a compact and interpretable subset of features for the HPS.

Favoring specificity over sensitivity is useful in settings where false-positive results need to be minimized and PPV needs to be high, such as expensive clinical trials. Here, with our VCOG HPS model, we report the highest PPVs for progression to AD from the MCI stage (up to 87.8%, adjusted for 33.6% prevalence of progressors) for models that included structural MRI and cognitive features, which are, importantly, modalities that are already widely used by clinicians. The present work could be used as a screening tool for recruitment in clinical trials that target participants with MCI who are likely to progress to dementia within 3 years. The implementation of an automated selection algorithm could also result in groups of participants with MCI with more homogeneous brain pathology. However, we note that high-confidence patients did not all present with significant amyloid burden (92.0% and 68.4% of high-confidence patients in ADNI1 and ADNI2, respectively; Fig. 4), which means that not all high-confidence individuals are likely to have prodromal AD, even when progressing to dementia.

When we trained our model with cognitive features only, tests for general cognition, memory, and executive function were chosen as the strongest predictors of AD dementia. Our COG HPS model thus supports previous research that reported general cognition, memory, and executive function as important neuropsychological predictors of dementia [7, 34, 59, 60]. Compared to the state-of-the-art multi-domain cognition-based predictive model, which reported 87.1% specificity and 81.8% PPV (77.5% when adjusted to 33.6% pMCI prevalence) [61], our COG HPS model achieved similar performance, reaching 87.5−95% specificity and 72.3−85.1% (adjusted) PPV. Because general cognition was the strongest feature in our model to predict progression, this supports previous findings that patients with MCI with deficits across multiple domains are at the highest risk for dementia [60, 62].

For our VBM model, we extracted a number of grey matter atrophy subtypes that recapitulated previously reported subtypes, namely, the medial temporal lobe and parietal-dominant subtypes [5, 49–51], which were associated strongly with a diagnosis of AD dementia. Weights for the parietal-dominant and medial temporal lobe subtypes (Subtypes 1 and 6 from Fig. 1b, respectively) contributed substantially to the HPS in the VBM model. The atrophy pattern of Subtype 6 is spatially similar to the spread of neurofibrillary tangles in Braak stages III and IV [63], which may support previous findings that τ aggregation mediates neurodegeneration [64]. The contributions of the parietal-dominant and medial temporal lobe subtypes in the VBM HPS model are also in line with previous works, which have reported that cortical thickness and volumes of the medial temporal lobes, inferior parietal cortex, and precuneus are strong predictors of progression to dementia [7, 11].

When combined with cognitive tests in the VCOG model, the structural subtypes were given marginal weights. This suggests some redundancy between atrophy and cognition, and that cognitive features have higher predictive power than structural features in the ADNI MCI sample. This conclusion is consistent with the observation that the COG model significantly outperformed the VBM model, similar to previous work [7]. Although cognitive markers were stronger features, the VCOG model assigned large negative weights for the structural Subtypes 3, which showed greater relative GMV in the temporal lobes, and 7, which showed greater relative GMV in the parietal, occipital, and temporal lobes. This means that these features were predictive of stable MCI in the VCOG model, in line with previous work showing that atrophy in these regions is predictive of progression to dementia [7, 11]. Furthermore, we demonstrated that combining MRI data with cognitive markers significantly improves upon a model based on MRI features alone. This result is again in line with the literature [7, 10], yet was shown for the first time for a model specifically trained for high PPV. Note that in the present study, the predictive model was trained exclusively on images acquired on 1.5T scanners from ADNI1. Good generalization to ADNI2 with 3T scanners demonstrates robustness of imaging structural subtypes across scanner makes.

The VCOG HPS might reflect a late disease stage. We looked at the ratio of early to late MCI participants in the ADNI2 sample (note that ADNI1 did not have participants with early MCI). Of the participants with MCI identified as high confidence by the VCOG model, 84.4% had late MCI, compared to a rate of 34.9% of participants with late MCI in the entire ADNI2 MCI sample (Supplementary Fig. S1). This approach may not be optimal for early detection of future cognitive decline. Training a model to classify MCI progressors and non-progressors to dementia could be done in order to capture future progressors in earlier preclinical stages (e.g., early MCI). Finally, we focused on structural MRI and neuropsychological batteries as features in our models due to their wide availability and established status as clinical tools. However, we believe that adding other modalities such as PET imaging, CSF markers, functional MRI, genetic factors, or lifestyle factors could result in higher predictive power, especially at earlier preclinical stages of AD.

## Conclusion

In summary, we found a subgroup of patients with MCI who share a signature of cognitive deficits and brain atrophy that put them at very high risk to progress from MCI to AD dementia within a time span of 3 years. We validated the signature in 2 separate cohorts that contained both stable patients with MCI and patients with MCI who progressed to dementia. The model was able to predict progression to dementia in patients with MCI with up to 93.1% PPV and up to 96.7% specificity. The signature was present in approximately half of all progressors, demonstrating that gains in PPV can be made by focusing on a homogeneous yet relatively common subgroup. Our model could potentially improve participant selection in clinical trials and identify individuals at a higher risk of AD dementia for early intervention in clinical settings.

## Availability of supporting source code and requirements

Project name: A signature of cognitive deficits and brain atrophy that is highly predictive of progression to Alzheimer's dementia Project home page: https://github.com/SIMEXP/vcog_hps_ad

Operating systems: Platform independent

Programming language: MATLAB/Octave, Python

Other requirements: SPM12, NeuroImaging Analysis Kit 0.18.1, Scikit-learn 0.18

License: MIT

## Availability of supporting data and materials

Simulated data of demographic variables and imaging measures are archived on Figshare [48]. Further supporting data and snapshots of our code are available in the *GigaScience* repository, Giga DB [65].

## Additional files

Supplementary methods. Details of the implementation for the SVM with RBF kernel, K nearest neighbours, random forest, and Gaussian Naive Bayes algorithms.

Table S1. pMCI vs sMCI performance metrics for VCOG HPS models with different number of VBM subtypes.

Table S2. Characteristics of high-confidence participants from the VBM, COG, and VCOG signatures in ADNI1 and ADNI2 MCI cohorts.

Figure S1. Percentage of late MCI ADNI2 participants within each HPS grouping (high confidence, low confidence, negative) across each highly predictive model (VCOG, VBM, COG).

Figure S2. Cognitive trajectories for individual MCI participants in ADNI1 and ADNI2 grouped by HPS classifications (high confidence, low confidence, negative) by the VCOG, COG, and VBM high confidence prediction models.

Figure S3. Coefficients of factors in the VCOG HPS models for a model featuring 3 VBM subtypes and a model featuring 10 VBM subtypes.

GIGA-D-18-00349_Original_Submission.pdf

GIGA-D-18-00349_Revision_1.pdf

GIGA-D-18-00349_Revision_2.pdf

GIGA-D-18-00349_Revision_3.pdf

Response_to_Reviewer_Comments_Original_Submission.pdf

Response_to_Reviewer_Comments_Revision_1.pdf

Response_to_Reviewer_Comments_Revision_2.pdf

Reviewer_1_Report_Original_Submission -- Yu-Dong Zhang 11/17/2018 Reviewed

Reviewer_1_Report_Revision_1 -- Yu-Dong Zhang 3/12/2019 Reviewed

Reviewer_2_Report_Original_Submission -- Hosung Kim 12/5/2018 Reviewed

Reviewer_2_Report_Revision_1 -- Hosung Kim 3/30/2019 Reviewed

Reviewer_3_Report_Original_Submission -- Abbas Babajani-Feremi 12/21/2018 Reviewed

Reviewer_3_Report_Revision_1 -- Abbas Babajani-Feremi 3/27/2019 Reviewed

Supplemental File

## Abbreviations

Aβ: amyloid beta; AD: Alzheimer's disease; ADAS13: Alzheimer's Disease Assessment Scale—Cognitive subscale (13 items); ADNI: Alzheimer's Disease Neuroimaging Initiative; APOE4: apolipoprotein E ε4; AUC: area under the curve; BNT: Boston Naming Test; CN: cognitively normal; COG: cognitive features; CSF: cerebrospinal fluid; GMV: grey matter volume; GNB: Gaussian naive Bayes; HPS: highly predictive signature; KNN: K nearest neighbours; MCI: mild cognitive impairment; MMSE: Mini Mental State Examination; MRI: magnetic resonance imaging; PET: positron emission tomography; pMCI: progressive mild cognitive impairment; PPV: positive predictive value; RBF: radial basis function; ROC: receiver operating characteristic; sMCI: stable mild cognitive impairment; SVM: support vector machine; TIV: total intracranial volume; VBM: voxel-based morphometry; VCOG: voxel-based morphometry and cognitive features.

## Competing interests

The authors declare that they have no competing interests.

## Funding

Data collection and sharing for this project was funded by the Alzheimer's Disease Neuroimaging Initiative (ADNI) (National Institutes of Health grant U01 AG024904) and DOD ADNI (Department of Defense award No. W81XWH-12-2-0012). ADNI is funded by the National Institute on Aging, the National Institute of Biomedical Imaging and Bioengineering, and through generous contributions from the following: AbbVie; Alzheimer's Association; Alzheimer's Drug Discovery Foundation; Araclon Biotech; BioClinica, Inc.; Biogen; Bristol-Myers Squibb Company; CereSpir, Inc.; Cogstate; Eisai, Inc.; Elan Pharmaceuticals, Inc.; Eli Lilly and Company; EuroImmun; F. Hoffmann-La Roche Ltd and its affiliated company Genentech, Inc.; Fujirebio; GE Healthcare; IXICO Ltd.; Janssen Alzheimer Immunotherapy Research & Development, LLC; Johnson & Johnson Pharmaceutical Research & Development, LLC.; Lumosity; Lundbeck; Merck & Co., Inc.; Meso Scale Diagnostics, LLC; NeuroRx Research; Neurotrack Technologies; Novartis Pharmaceuticals Corporation; Pfizer, Inc.; Piramal Imaging; Servier; Takeda Pharmaceutical Company; and Transition Therapeutics. The Canadian Institutes of Health Research is providing funds to support ADNI clinical sites in Canada. Private sector contributions are facilitated by the Foundation for the National Institutes of Health (www.fnih.org). The grantee organization is the Northern California Institute for Research and Education, and the study is coordinated by the Alzheimer's Therapeutic Research Institute at the University of Southern California. ADNI data are disseminated by the Laboratory for Neuro Imaging at the University of Southern California.

The computational resources used to perform the data analysis were provided by Compute Canada (www.computecanada.org). This project was funded by NSERC grant RN000028 and the Canadian Consortium on Neurodegeneration in Aging (CCNA, www.ccna-ccnv.ca), through a grant from the Canadian Institutes of Health Research and funding from several partners including SANOFI-ADVENTIS R&D. A.T. was supported by a bursary from the Centre de recherche de l'institut universitaire de gériatrie de Montréal and the Courtois foundation. C.D. was supported by a salary award from the Lemaire foundation and Courtois foundation. P.B. was supported by a salary award from Fonds de recherche du Québec–Santé and the Courtois foundation.

## Authors’ contributions

Writing original draft: A.T., P.B.; review and editing: A.T., C.D., J.B., P.B.; supervision: J.B., P.B.; conceptualization: A.T., C.D., P.O., P.B.; software: A.T., C.D., Y.I.M., S.U., P.O., H.S.; methodology: C.D., Y.I.M., S.U., P.O., P.B.; formal analysis and investigation: A.T.; visualization: A.T.; data curation: A.T., H.S.; funding acquisition: P.B.; resources: P.B.
