# Supplementary material for: A highly predictive signature of cognition and brain atrophy for progression to Alzheimer's dementia
Source: Gigascience. 2019 May 11;8(5):giz055. doi: 10.1093/gigascience/giz055 (PMC6511068; doi:10.1093/gigascience/giz055)
Supplement: GIGA-D-18-00349_Original_Submission.pdf [file giz055_giga-d-18-00349_original_submission.pdf]

// GigaScience

A highly predictive signature of cognition and brain atrophy for progression to Alzheimer's dementia

--Manuscript Draft--

**Manuscript Number:**	GIGA-D-18-00349
**Full Title:**	A highly predictive signature of cognition and brain atrophy for progression to Alzheimer's dementia
**Article Type:**	Research
**Funding Information:**	Canadian Network for Research and Innovation in Machining Technology, Natural Sciences and Engineering Research Council of Canada (CA) (RN000028) — Dr Pierre Bellec<br>Courtois Foundation (n/a) — Dr Pierre Bellec<br>Lemaire Foundation (n/a) — Dr Pierre Bellec
**Abstract:**	Patients with mild cognitive impairment (MCI) are at risk of progressing to Alzheimer's dementia, yet only a fraction of them do. We explore here whether a very high-risk MCI subgroup can be identified using cognitive assessments and structural neuroimaging. A multimodal signature of Alzheimer's dementia was first extracted using machine learning tools in the ADNI1 sample, and was comprised of cognitive deficits across multiple domains as well as atrophy in temporal, parietal and occipital regions. We then validated the predictive value of this signature on two MCI cohorts. In ADNI1 (N=235), the presence of the signature predicted progression to dementia over three years with 80.4% positive predictive value, adjusted for a "typical" MCI baseline rate of 33% (95.6% specificity, 55.1% sensitivity). These results were replicated in ADNI2 (N=235), with 87.8% adjusted positive predictive value (96.7% specificity, 47.3% sensitivity). Our results demonstrate that, even for widely used markers, marked improvement in positive predictive value over the literature can be achieved by focusing on a subgroup of individuals with similar brain characteristics. The signature can be readily applied for the enrichment of clinical trials.
**Corresponding Author:**	Angela Tam, PhD<br>Centre de recherche de l'institut universitaire de gériatrie de Montréal<br>Montreal, Quebec CANADA
**Corresponding Author Secondary Information:**	
**Corresponding Author's Institution:**	Centre de recherche de l'institut universitaire de gériatrie de Montréal
**Corresponding Author's Secondary Institution:**	
**First Author:**	Angela Tam, PhD
**First Author Secondary Information:**	
**Order of Authors:**	Angela Tam, PhD<br>Christian Dansereau, PhD<br>Yasser Iturria-Medina, PhD<br>Sebastian Urchs, MSc<br>Pierre Orban, PhD<br>Hanad Sharmarke, BSc<br>John Breitner, MD<br>Pierre Bellec, PhD

Powered by Editorial Manager® and ProduXion Manager® from Aries Systems Corporation

Order of Authors Secondary Information:	
Additional Information:	
Question	Response
Are you submitting this manuscript to a special series or article collection?	No
**Experimental design and statistics**<br><br>Full details of the experimental design and statistical methods used should be given in the Methods section, as detailed in our Minimum Standards Reporting Checklist. Information essential to interpreting the data presented should be made available in the figure legends.<br><br>Have you included all the information requested in your manuscript?	Yes
**Resources**<br><br>A description of all resources used, including antibodies, cell lines, animals and software tools, with enough information to allow them to be uniquely identified, should be included in the Methods section. Authors are strongly encouraged to cite Research Resource Identifiers (RRIDs) for antibodies, model organisms and tools, where possible.<br><br>Have you included the information requested as detailed in our Minimum Standards Reporting Checklist?	Yes
**Availability of data and materials**<br><br>All datasets and code on which the conclusions of the paper rely must be either included in your submission or deposited in publicly available repositories (where available and ethically appropriate), referencing such data using a unique identifier in the references and in the “Availability of Data and Materials” section of your manuscript.	Yes

Have you have met the above requirement as detailed in our Minimum Standards Reporting Checklist?	

Powered by Editorial Manager® and ProduXion Manager® from Aries Systems Corporation

Manuscript
Click here to view linked References
Click here to access/download;Manuscript;gigascience_vcog_paper_v2.pdf

A highly predictive signature of cognition and brain atrophy for progression to Alzheimer's dementia

Short running title: Signature of future Alzheimer’s dementia

Angela Tam[1,2,3,]†, Christian Dansereau[1,4], Yasser Itturia-Medina[3], Sebastian Urchs[3], Pierre Orban[1,5,6], Hanad Sharmarke[1], John Breitner[2,3], Pierre Bellec[1,4,]†, for the Alzheimer’s Disease Neuroimaging Initiative*

1. Centre de Recherche de l'Institut Universitaire de Gériatrie de Montréal, Montréal, CA
2. Douglas Hospital Research Centre, McGill University, Montréal, CA
3. McGill University, Montréal, CA
4. Département d'Informatique et de recherche opérationnelle, Université de Montréal, Montréal, CA
5. Centre de Recherche de l'Institut Universitaire en Santé Mentale de Montréal, Montréal, CA
6. Département de Psychiatrie, Université de Montréal, Montréal, CA

*Data used in preparation of this article were obtained from the Alzheimer's Disease Neuroimaging Initiative (ADNI) database (adni.loni.usc.edu). As such, the investigators within the ADNI contributed to the design and implementation of ADNI and/or provided data but did not participate in analysis or writing of this report. A complete listing of ADNI investigators can be found at: http://adni.loni.usc.edu/wp-content/uploads/how_to_apply/ADNI_Acknowledgement_List.pdf

†Corresponding authors:
angela.tam@mail.mcgill.ca
pierre.bellec@criugm.qc.ca
4545 Queen Mary Rd
Montreal QC
Canada H3W 1W6

Abstract

Patients with mild cognitive impairment (MCI) are at risk of progressing to Alzheimer's dementia, yet only a fraction of them do. We explore here whether a very high-risk MCI subgroup can be identified using cognitive assessments and structural neuroimaging. A multimodal signature of Alzheimer's dementia was first extracted using machine learning tools in the ADNI1 sample, and was comprised of cognitive deficits across multiple domains as well as atrophy in temporal, parietal and occipital regions. We then validated the predictive value of this signature on two MCI cohorts. In ADNI1 (N=235), the presence of the signature predicted progression to dementia over three years with 80.4% positive predictive value, adjusted for a "typical" MCI baseline rate of 33% (95.6% specificity, 55.1% sensitivity). These results were replicated in ADNI2 (N=235), with 87.8% adjusted positive predictive value (96.7% specificity, 47.3% sensitivity). Our results demonstrate that, even for widely used markers, marked improvement in positive predictive value over the literature can be achieved by focusing on a subgroup of individuals with similar brain characteristics. The signature can be readily applied for the enrichment of clinical trials.

Keywords

Alzheimer's disease, mild cognitive impairment, machine learning, neuroimaging, cognition

Introduction

Alzheimer's disease (AD), a leading cause of dementia, is marked by the abnormal accumulation of amyloid β (Aβ) and hyperphosphorylated tau proteins in the brain, which leads to widespread neurodegeneration. AD has a long prodromal phase, and it has been difficult to predict which individuals will decline and experience AD dementia. While mild cognitive impairment (MCI) is often considered a prodromal stage of AD, only a fraction (up to 36%) of MCI patients will develop dementia within two years [1]. Identifying MCI patients who will progress to AD dementia with enough specificity has been a challenge for clinical trials, where the inclusion criteria for MCI subjects have had low to moderate positive predictive value (PPV) [2]. This lack of prognostic power may be due to individual variability. Different clinical phenotypes have been described where patients will exhibit distinct cognitive deficits [3]. Previous work has also characterized neuropathological subtypes based on the distribution of neurofibrillary tangles [4], which correspond well to distinct patterns of brain atrophy [5]. Different subtypes of brain atrophy have also been associated with different rates of progression to dementia [6]. The implications for prognosis are profound: only a subgroup of patients will have clinical trajectories that can be reliably predicted. We therefore propose to identify a subset of individuals with a homogenous signature of brain atrophy and cognitive deficits who will progress to AD dementia with high precision.

There is a large field focused on using machine learning to automatically detect MCI patients who will progress to AD dementia based on imaging and cognitive features. For models combining structural MRI and cognition, in terms of accuracy, the best has been reported at 79% (76% specificity, 83% sensitivity) [7]. The state-of-the-art, which used Aβ positron emission tomography scans, has so far achieved 82% accuracy (87% specificity, 71% sensitivity) [8]. Note that accuracy is defined as the proportion of subjects that were correctly identified, either as positives or negatives. PPV, on the other hand, is defined as the proportion of true positives out of all subjects that were classified as positive. A model can have high accuracy yet moderate PPV if the proportion of true positives is low relative to true negatives. In the case of predicting incipient AD, relatively few MCI subjects progress to dementia. Despite promising accuracy, the PPVs of models predicting AD progression remain moderate, ranging from 50 to 75% across the literature [9]. This implies that up to half of subjects who were identified as progressors by published algorithms did not actually progress

to dementia. It seems that by focussing our efforts on improving accuracy, we have a reached a plateau in generating models with good precision. We therefore aimed to create a predictive model that was optimized for high PPV.

In this work, we developed a multimodal signature that is highly predictive of progression to AD dementia in a subgroup of MCI patients, based on cognition and subtypes of grey matter atrophy. We also aimed to evaluate the complementarity of features derived from cognition and atrophy patterns. Although this has been extensively studied for prognosis of dementia in a general MCI population, the complementarity of these measures is not documented in the specific context of a high risk signature. We applied a cluster analysis on structural magnetic resonance images to identify subtypes of brain atrophy in a sample containing both patients with AD dementia and cognitively normal (CN) individuals. We then used a two-step machine learning algorithm [9] to train a model to identify three signatures that were highly predictive of AD dementia: 1) an anatomical signature, 2) a cognitive signature, 3) a multimodal anatomical and cognitive signature. After identifying MCI patients carrying these signatures, we examined cognitive decline, Aβ and tau burden, and progression to dementia in these individuals to explore whether a highly predictive signature represented a prodromal stage of AD. We analysed whether these three signatures identified separate subgroups of subjects and how they performed against each other in terms of PPV, specificity, and sensitivity at identifying progressors.

Materials and methods

Data

Data used in the preparation of this article were obtained from the Alzheimer's Disease Neuroimaging Initiative (ADNI) database (adni.loni.usc.edu). The ADNI was launched in 2003 as a public-private partnership, led by Principal Investigator Michael W. Weiner, MD. The primary goal of ADNI has been to test whether serial magnetic resonance imaging (MRI), positron emission tomography (PET), other biological markers, and clinical and neuropsychological assessment can be combined to measure the progression of mild cognitive impairment (MCI) and early Alzheimer's disease (AD). For up-to-date information, see www.adni-info.org.

We took baseline T1-weighted MRI scans from the ADNI1 (228 CN, 397 MCI, 192 AD) and ADNI2 (218 CN, 354 MCI, 103 AD) studies. For a detailed description of MRI acquisition details, see http://adni.loni.usc.edu/methods/documents/mri-protocols/. All subjects gave informed consent to participate in these studies, which were approved by the research ethics committees of the institutions involved in data acquisition. Consent was obtained for data sharing and secondary analysis, the latter being approved by the ethics committee at the CRIUGM. For the MCI groups, each individual must have had at least 36 months of follow-up for inclusion in our analysis. We also further stratified the MCI groups into stable (sMCI), who never received any change in their diagnosis, and progressors (pMCI), who received a diagnosis of AD dementia within 36 months of follow-up. pMCI who progressed to AD dementia after 36 months were excluded. After applying these inclusion/exclusion criteria, we were left with 280 and 268 eligible MCI subjects in ADNI1 and ADNI2 respectively.

Structural features from voxel-based morphometry

Images were processed with the NeuroImaging Analysis Kit (NIAK) version 0.18.1 (https://hub.docker.com/r/simexp/niak-boss/), the MINC toolkit (http://bic-mni.github.io/) version 0.3.18, and SPM12 (http://www.fil.ion.ucl.ac.uk/spm/software/spm12/) under CentOS with Octave (http://gnu.octave.org) version 4.0.2. Preprocessing of MRI data was executed in parallel on the Cedar supercomputer (https://docs.computecanada.ca/wiki/Cedar), using the Pipeline System for Octave and Matlab (PSOM) [10]. Each T1 image was linearly co-registered to the Montreal Neurological Institute (MNI) ICBM152 stereotaxic symmetric template [11], using the CIVET pipeline [12], and then re-oriented to the AC-PC line. Each image was segmented into grey matter, white matter, and CSF probabilistic maps. The DARTEL toolbox [13] was used to normalize the grey matter segmentations to a predefined grey matter template in MNI152 space. Each map was modulated to preserve the total amount of signal and smoothed with with a 8 mm isotropic Gaussian blurring kernel. After quality control of the normalized grey matter segmentations, we were left with 621 subjects in ADNI1 (out of 700, 88.7% success rate) and 515 subjects in ADNI2 (out of 589, 87.4% success rate).

We extracted subtypes to characterize variability of grey matter distribution with the CN and AD samples from ADNI1. In order to reduce the impact of factors of no interest that

may have influenced the clustering procedure, we regressed out age, sex, mean grey matter volume (GMV), and total intracranial volume (TIV), using a mass univariate linear regression model at each voxel. We then derived a spatial Pearson's correlation coefficient between all pairs of individual maps after confound regression. This defined a subject x subject (377 x 377) similarity matrix which was entered into a Ward hierarchical clustering procedure (Figure 1a). Based on visual inspection of the similarity matrix, we identified 7 subgroups (Figure 1b). Each subtype was defined as the average map of each subgroup. For each subject, we computed spatial correlations between their map and each subtype, which we call weights (Figure 1a). The weights formed a n subject x n subtypes matrix, which was included in the feature space for all predictive models including VBM throughout this work. As in our previous works [9,14], we chose to use weights, which can be interpreted as continuous measures for subtype affinity, over discrete subtype membership because the latter is less informative as most individuals express similarity to multiple subtypes [15]. Note that although we chose to present our findings with 7 subtypes, we examined how the number of subtypes may impact our subsequent predictions. There was no significant difference in model performance when we changed the number of subtypes (see Table S1 in supplementary material).

Cognitive features

We took baseline neuropsychological scores for each subject from several cognitive domains: memory from the composite score ADNI-MEM [16], executive function from the composite score ADNI-EF [17], language from the Boston Naming Test (BNT), visuospatial from the clock drawing test, and global cognition from the Alzheimer's Disease Assessment Scale-Cognitive (ADAS13). We chose measures that span multiple cognitive domains as it has been suggested that the use of a combination of neuropsychological measures is likely the best approach to predicting incipient dementia [18]. These scores were included as features for the predictive models involving cognition. Thirteen subjects across both ADNI1 and ADNI2 (8 AD, 5 MCI) had to be excluded due to missing values in their cognitive assessments. See Table 1 for demographic information of subjects who were included in analyses.

Prediction of easy AD dementia cases in ADNI1

We trained a linear support vector machine (SVM) model with a linear kernel, as implemented by Scikit-learn [19] version 0.18 to classify AD vs CN from ADNI1 to get a baseline prediction accuracy. A tenfold cross-validation loop was used to estimate the performance of the trained model. Classes were balanced inversely proportional to class frequencies in the input data for the training. A nested cross-validation loop (stratified shuffle split with 50 splits and 20% test size) was used for the grid search of the hyperparameter *C* (grid was 10^{-2} to 10^{1} with 15 equal steps). We randomly selected subsamples of the dataset (retaining 50% of participants in each subsample) to replicate the SVM training 500 times. For each 50% subsample, a separate SVM model was trained to predict AD or CN in ADNI1. Predictions were made on the remaining 50% of the sample that was not used for training. For each subject, we calculated a hit probability defined as the frequency of correct classification across all SVM replications in which the test set contained that subject. Easy AD cases were defined as individuals with 100% hit probabilities with the AD label. Next, we trained a logistic regression classifier [20], with L1 regularization on the coefficients, to predict the easy AD cases. A stratified shuffle split (500 splits, 50% test size) was used to estimate the performance of the model for the grid search of the hyperparameter *C* (grid was 10^{-2} to 10^{1} with 15 equal steps). See [9] for more information about this two-step prediction. We used the whole CN and AD sample from ADNI1 to obtain three highly predictive signatures (HPS), one using VBM subtypes (VBM only), one using cognitive features (COG only), and one using the combination of VBM and cognitive features (VCOG). In all three signatures, age, sex, mean GMV, and TIV were also included as features.

Prediction of progression to AD dementia from the MCI stage in ADNI1

The logistic regression trained on AD vs CN was used to identify MCI patients who have a HPS of AD dementia in ADNI1. We re-trained our models on AD vs CN after optimizing our hyperparameters (resampling size and resampling ratio) in order to maximize specificity and PPV while keeping a minimum of 30% sensitivity for our classification of sMCI (n=89) vs pMCI (n=155) in ADNI1. This was done for all three signatures. In brief, we

used the AD and CN sample from ADNI1 as a training set, the MCI subjects from ADNI1 as a validation set, and ADNI2 served as our test set.

Statistical test of differences in model performance

We used Monte-Carlo simulations to generate confidence intervals on the performance (i.e. PPV, specificity and sensitivity) of both base SVM and HPS models for their predictions of AD vs CN and pMCI vs sMCI. Taking the observed sensitivity and specificity, and using similar sample sizes to our experiment, we replicated the number of true and false positive detection 100000 times using independent Bernoulli variables, and derived replications of PPV, specificity and sensitivity. By comparing these replications to the sensitivity, specificity and PPV observed in both models, we estimated a p-value for differences in model performance [21]. A p-value smaller than 0.05 was interpreted as evidence of a significant difference in performance, and 0.001 as strong evidence. We also used this approach to compare the performance of the combined features (VCOG) to the models containing VBM features or cognitive features only. Note that, based on our hypotheses regarding the behaviour of the HPS model, the tests were one-sided for increased specificity and PPV, and one-sided for decreased sensitivity.

Statistical tests of association of progression, AD biomarkers, and risk factors in HPS+ MCI subjects

Based on the classifications resulting from the base SVM and HPS models, we separated the MCI subjects into three different groups: 1) HPS+, subjects who were selected by the HPS model as hits, 2) Non-HPS+, subjects who were selected by the base SVM model as hits but were not selected by the HPS model, and 3) Negative, subjects who were not selected as hits by either algorithm.

We tested statistically if the HPS+ subgroup was enriched for progression to dementia, APOE4 carriers, females, and subjects who were positive for Aβ and tau pathology. Positivity of AD pathology was determined by CSF measurements of Aβ 1-42 peptide and total tau with cut-off values of less than 192 pg/mL and greater than 93 pg/mL respectively [22]. We implemented Monte-Carlo simulations, where we selected 100000 random subgroups out of the original MCI sample. By comparing the proportion of progressors,

APOE4 carriers, females, Aβ-positive, and tau-positive subjects in these null replications to the actual observed values in the HPS subgroup, we estimated a p-value [21] (one sided for increase). A p-value smaller than 0.05 was interpreted as evidence of a significant enrichment, and 0.001 as strong evidence.

One-way ANOVAs were used to evaluate differences between the HPS groupings with respect to age. Post-hoc Tukey's HSD tests were done to assess pairwise differences among the three classes (HPS+, Non-HPS+, Negative). These tests were implemented in Python with the SciPy library [23] version 0.19.1 and StatsModels library [24] version 0.8.0.

To explore the impact of HPS grouping on cognitive trajectories, linear mixed effects models were performed to evaluate the main effects of and interactions between the HPS groups and time on ADAS13 scores up to 36 months of follow-up. The models were first fit with a random effect of participant and then were fit with random slopes (time | participant) if ANOVAs comparing the likelihood ratio suggested a significant improvement in model fit. All tests were performed separately on the ADNI1 and ADNI2 datasets. These tests were implemented in R version 3.3.2 with the library nlme version 3.1.128 [25].

Public code and data availability

The code used in this experiment is available on a GitHub repository (https://github.com/SIMEXP/vcog_hps_ad) and zenodo (https://doi.org/10.5281/zenodo.1444081).

We shared a notebook replicating all the machine learning experiments, starting after the generation of VBM subtypes. However, in order to protect the privacy of the study participants, we could not share individual subtype weights alongside other behavioural data and diagnostic information. We thus created parametric (Gaussian) bootstrap simulations, based on group statistics alone, that will allow interested readers to replicate results similar to those presented in this manuscript, using the exact same code and computational environment that were used on real data, but with purely synthetic data instead. The notebook can be executed online via the binder platform (http://mybinder.org), and runs into a docker container (https://mybinder.org/v2/gh/HanadS/vcog_hps_ad/master?filepath=%2Fvcog_hpc_prediction_simulated_data.ipynb), built from a configuration file that is available on GitHub

(https://github.com/SIMEXP/vcog_hps_ad/blob/master/Dockerfile). The container itself is available on Docker Hub (https://hub.docker.com/r/simexp/vcog_hps_ad/). The simulated data was archived on figshare (https://figshare.com/articles/Simulated_cognitive_and_structural_MRI_data_from_ADNI/7132757).

The simulation included the following 16 variables: age, sex, mean grey matter volume, total intracranial volume, 5 cognitive assessment scores and 7 VBM subtype weights from both ADNI1 and ADNI2. Subjects that had missing values for these variables were discarded from the simulation, leaving N=1115 subjects. We stratified the population into 12 subgroups: the four clinical labels (AD, pMCI, sMCI, CN), each further subdivided by the three prediction subclasses identified in this paper (Negative, Non-HPS+, HPS+). For each subgroup, we estimated the average and covariance matrices between the 16 variables of interest. We then generated a number of multivariate normal data points that matched the number of subjects found in each subgroup, using the empirical mean and covariance matrix of each subgroup. Finally, the range of the simulated data was clipped to the range of the original real data, and the simulated sex data points were binarized by nearest neighbour.

The statistics from the predictive model in the original implementation are similar to that of the simulated data. The model predicted the progression of dementia from MCI in ADNI1 with a PPV of 93.1% (specificity of 93.2%) on real data. This coincides with a 93.3% PPV (specificity of 94.3%) that we get when using the simulated data. Similarly, with the ADNI2 dataset the model achieved a 81.3% PPV (specificity of 96.7%) from the real data and a 75.7% PPV (specificity of 95.0%) from the simulated data.

Results

Prediction of AD dementia vs cognitively normal individuals

The SVM model trained using the VCOG features achieved 94.5% PPV (95.6% specificity, 93.9% sensitivity) when classifying AD vs CN in ADNI1. Such high performance was expected given the marked cognitive deficits associated with clinical dementia. COG features only actually reached excellent performance as well (97.6% PPV, 98.0% specificity, 96.4% sensitivity), while using VBM features only yielded markedly lower performances (86.4% PPV, 89.3% specificity, 79.6% sensitivity) (see Figure 2a). Note that the performance

metrics in ADNI1 were estimated through cross-validation, and represent an average performance for several models trained on different subsets of ADNI1. We then trained a model on all of ADNI1, and estimated its performance on an independent dataset, ADNI2. Using VCOG predictors, the ADNI1 model reached 92.0% PPV (96.3% specificity, 92.0% sensitivity), when applied on ADNI2 AD vs CN data. Again the performance was comparable with COG predictors only (92.2% PPV, 96.3% specificity, 94.3% sensitivity), and lower performance with VBM features only (57.3% PPV, 79.8% specificity, 56.7% sensitivity) (see Figure 2a). Note that PPV is dependent on the proportion of patients and controls for a given sensitivity and specificity. Since the ADNI2 sample had a substantially smaller proportion of AD subjects compared to ADNI1, the resulting PPV was reduced. When we adjusted the baseline rate of AD subjects in ADNI2 to the same rate in ADNI1, the PPVs were 95.2%, 95.3%, and 70.2% for the VCOG, COG, and VBM models respectively.

Identification of easy AD cases for prediction

The VCOG HPS model achieved 99.2% PPV (99.5% specificity, 77.6% sensitivity) in classifying easy AD subjects in ADNI1. These performance scores were estimated by cross-validation of the entire two-stage process (training of SVM, estimation of hit probability, identification of HPS). However, the hyperparameters of the two-stage model were optimized on classifying pMCI vs sMCI in ADNI1, as described below. We next trained a single model on all of ADNI1, which we applied on an independent sample (ADNI2). The ADNI1 AD VCOG HPS model reached 98.6% PPV (99.5% specificity, 79.5% sensitivity) on ADNI2. As was previously observed with the conventional SVM analysis, the VCOG HPS model had similar performance to the COG HPS model (ADNI1: 100% PPV, 100% specificity, 87.3% sensitivity; ADNI2: 98.7% PPV, 99.5% specificity, 88.6% sensitivity), and outperformed the VBM HPS model (ADNI1: 92.3% PPV, 96.1% specificity, 54.6% sensitivity; ADNI2: 65.2% PPV, 91.5% specificity, 33.3% sensitivity), see Figure 2a. When adjusted to the same baseline rate of AD subjects as ADNI1, the PPVs in ADNI2 were 99.2%, 99.3%, and 76.7% for the VCOG, COG, and VBM HPS models respectively.

The HPS models consistently outperformed the base SVM classifiers with respect to specificity ($p<0.001$) in the classifications of AD vs CN and pMCI vs sMCI in both ADNI1 and ADNI2, regardless of the features that the models contained. The HPS also had greater PPV ($p<0.05$) adjusted for a typical prevalence of 33.6% pMCI in a given sample of MCI

subjects [26]. However, these increases in specificity and PPV for the HPS model came at a significant cost of reduced sensitivity compared to the base classifier, across all models in both ADNI1 and ADNI2 ($p<0.05$) (Figure 2). Note that this shift towards lower sensitivity and higher specificity and PPV could be achieved by adjusting the threshold of the SVM analysis (see ROC analysis, Figure S1 in supplementary material), and is not unique to the two-stage procedure we implemented.

High confidence prediction of progression to AD dementia

When using the full VCOG features, 87 MCI patients were selected as HPS+ in ADNI1, out of which 81 (93.1% PPV) were pMCI within 36 months of follow-up. This represented a large, significant increase over the baseline rate of progressors in the entire ADNI1 MCI sample (37.4%) ($p<0.001$). This was also a significant increase over the SVM's predictions, where 83.9% of subjects that it had labeled as hits were true progressors ($p<0.001$). When adjusted to a 33.6% baseline rate of progressors, more typical of MCI populations, the PPV of HPS+ for prognosis of dementia was 80.4% (93.2% specificity, 55.1% sensitivity).

We replicated these analyses in the MCI sample from ADNI2 (N=235). Using VCOG features, 32 subjects were identified as HPS+, 26 of which progressed to AD dementia within 36 months follow-up (81.2% PPV, specificity of 96.7%, sensitivity of 47.3%, 87.8% PPV adjusted to a 33.6% baseline rate). This represented a significantly higher prevalence than the 30.6% baseline rate in the entire ADNI2 MCI cohort ($p<0.001$). This was also a significant increase over the SVM's predictions, where 67.8% of subjects it had labeled as hits were true progressors ($p<0.001$). As expected, the HPS+ model had lower sensitivity and higher specificity/PPV than the baseline model, both in ADNI1 and ADNI2, and for all three feature sets (VBM, COG and VCOG), see Figure 2b. The VCOG features also lead to higher PPV than VBM and COG features taken independently, both in ADNI1 and ADNI2. That increase was large and significant between VCOG and VBM (up to 17%) and marginal and non-significant between VCOG and COG (up to 8%), see Figure 2b.

Characteristics of MCI subjects with a highly predictive VCOG signature of AD

HPS+ MCI subjects with the VCOG signature were more likely to be progressors (Figure 3a) compared to non-HPS+ subjects and negative subjects (ADNI1: $p<0.001$; ADNI2: $p<0.001$). HPS+ MCI subjects were also more likely to be APOE4 carriers (Figure 3b) (ADNI1: $p<0.005$; ADNI2: $p<0.05$). There was no difference in sex across the HPS groupings in the MCI subjects of either the ADNI1 or ADNI2 cohorts (Figure 3c). This was consistent with the whole sample, where there were equal proportions of progressors across both sexes in each dataset (ADNI1: $\chi^2=0.015$, $p=0.90$; ADNI2: $\chi^2=0.0002$, $p=0.99$). The HPS+ class was also significantly enriched for Aβ-positive subjects in ADNI1 ($p<0.05$). However, this result was not replicated in the ADNI2 MCI subjects (Figure 3d). Similarly with tau, we found a significant increase in tau-positive subjects in the HPS+ group of ADNI1 ($p<0.05$), but not in ADNI2 (Figure 3e). We found a significant age difference across the HPS classes in ADNI2 ($F=5.68$, $p<0.005$), where the HPS+ subjects were older than the Negative subjects by a mean of 4.4 years. However, age did not differ across the HPS classes in ADNI1 (Figure 3f). Finally, HPS+ subjects had significantly steeper cognitive declines compared to the Non-HPS+ and Negative groups (Figure 3g): there were significant interactions between the HPS groupings and time in ADNI1: (HPS+ $\beta=-0.147$, $t=-7.56$, $p<0.001$; Non-HPS+ $\beta=-0.055$, $t=-2.46$, $p<0.05$) and ADNI2 (HPS+ $\beta=-0.194$, $t=-8.69$, $p<0.001$; Non-HPS+ $\beta=-0.072$, $t=-3.31$, $p=0.001$). The HPS+ in ADNI1 and ADNI2 respectively gained 1.8 and 2.3 more points each year on the ADAS13 compared to the Non-HPS+ and Negative groups. Note that higher scores on the Alzheimer's Disease Assessment Scale - Cognitive subscale (13 items) (ADAS13) represent worse cognitive function.

COG, VBM and VCOG signatures

The COG signature was mainly driven by scores from the ADAS13, which measures overall cognition, ADNI-MEM, a composite score that measures memory [16], and ADNI-EF, a composite score that measures executive function [27] (coefficients were 5.49, -4.80 and -2.50 respectively). In this model, sex, age, mean GMV, and TIV contributed very little, relative to the cognitive features (Figure 4b). Note that these coefficients should be interpreted as pseudo z-scores as the features had been normalized to zero mean and unit variance.

Almost all grey matter subtypes contributed to the VBM signature. Mean GMV, subtype 1 and subtype 6 had the highest weights in the model (coefficients were -5.07, 4.87, and 3.98 respectively) (Figure 4c). Subtype 1 was characterized by reduced relative GMV in the occipital, parietal and posterior temporal lobes. Subtype 6 was characterized by reduced relative GMV in the temporal lobes, notably the medial temporal regions. We had anticipated the larger contribution of these two subtypes as they have been described in previous AD subtyping work [5,28–30]. Diagnosis (CN, sMCI, pMCI, AD) accounted for a substantial amount of variance in these subtype weights (subtype 1: F=8.51, p<0.001; subtype 2: F=34.27, p<0.001). Post-hoc t-tests showed AD subjects had significantly higher weights compared to CN (Figure 1), making these subtypes associated with a diagnosis of AD (subtype 1: t=2.88, p<0.05; subtype 6: t=7.68, p<0.001).

The ADAS13, memory (ADNI-MEM) and executive function (ADNI-EF) scores contributed the most to the VCOG HPS signature (coefficients were 6.27, -7.43 and -3.95 respectively, Figure 4a). Of the VBM features, subtypes 2, 3 and 7 contributed the most to the signature (coefficients were 1.36, -2.12 and -2.83 respectively). Subtypes 1 and 6, which had the highest positive weights in the VBM model, were given marginal weights in the VCOG model, which is potentially indicative of redundancy with COG features. Subtype 2, which was associated with AD, was characterized by greater relative GMV in medial parts of the parietal and occipital lobes and the cingulate cortex, but less GMV everywhere else. Subtype 3 was characterized by greater relative GMV in the temporal lobes, insula and striatum. Subtype 7, which was associated with healthy controls, was characterized by greater relative GMV in the parietal, occipital and temporal lobes. Note that the weights for subtypes 3 and 7 were negative in the model, which means that predicted AD and pMCI cases had brain atrophy patterns that were spatially dissimilar to those subtypes.

Discussion

We developed a highly precise and specific MRI and cognitive-based model to predict AD dementia. Our two-stage predictive model reached 93.2% specificity and 93.1% PPV (80.4% when adjusted for 33.6% prevalence of progressors) in ADNI1 when classifying progressor vs stable MCI patients (within 3 years follow-up). We replicated these results in ADNI2 where the model reached 96.7% specificity and 81.2% PPV (87.8% adjusted PPV).

With respect to specificity and PPV, these results are a substantial improvement over previous works combining structural MRI and cognition on the same prediction task, that have reported up to 76% specificity and 65% PPV (adjusted for 33.6% prevalence of progressors) [7]. We also report the highest PPV compared to the current state-of-the-art predictive model using Aβ PET scans, which reported 74% (adjusted) PPV [8]. Finally, our results also reproduced our past work which developed a model that optimizes specificity and PPV [9]. Our performance is close to the 90% PPV reported by [9], which used a combination of structural and functional MRI measures and a two-stage predictive model, with the limitation of a smaller sample size (N=56 MCI patients) due to limited availability of functional MRI data in ADNI.

An ideal model to predict conversion to AD dementia would have both high sensitivity and specificity. However, the pathophysiological heterogeneity of clinical diagnosis will prevent highly accurate prediction linking brain features to clinical trajectories. We argue that, faced with heterogeneity, it is necessary to sacrifice sensitivity to focus on a subgroup of individuals with similar brain abnormalities. The high specificity of our two-stage model indeed came at a cost of reduced sensitivity (55.1% in ADNI1 and 47.3% in ADNI2 for classifying pMCI vs sMCI), which is much lower than sensitivity values of 64%-95% reported by other groups [9]. The two-stage procedure, based on prediction stability, did not offer gains compared to a simpler SVM model, if the threshold of the SVM model could be selected *a priori* to match the specificity of the two-stage procedure (see ROC curves in Figure S1 in supplementary material). The two-stage prediction model offered the advantage of a principled approach to train the prediction model in a high-specificity regime, based on stability. The choice of a L1 regularized logistic regression also lead to a compact and interpretable subset of features for the HPS.

Favoring specificity over sensitivity is useful in settings where false positives need to be minimized and PPV needs to be high, such as expensive clinical trials. Here, with our HPS VCOG model, we report the highest PPVs for progression to AD from the MCI stage (up to 87.8%, adjusted for 33.6% prevalence of progressors). Importantly, the proposed HPS+ model used tools that are already widely used by clinicians. The present work could be used as a screening tool for recruitment in clinical trials that target MCI subjects who are likely to progress to dementia within three years. The implementation of an automated selection algorithm could also result in groups of MCI subjects with more homogeneous brain

pathology. However, we note that HPS+ subjects did not all present with significant amyloid burden (92.0% and 68.4% of HPS+ subjects in ADNI1 and ADNI2 respectively, Figure 3), which means that not all HPS+ individuals are likely to have prodromal AD, even when progressing to dementia.

When we trained our model with cognitive features only, tests for general cognition, memory, and executive function were chosen as the strongest predictors of AD dementia. Our COG HPS model thus supports previous research that reported general cognition, memory, and executive function as important neuropsychological predictors of dementia [7,18,31,32]. Compared to the state-of-the-art multi-domain cognition-based predictive model, which reported 87.1% specificity and 81.8% PPV (77.5% when adjusted to 33.6% pMCI prevalence) [33], our COG HPS model achieved similar performance reaching between 87.5%-95% specificity and 72.3%-85.1% (adjusted) PPV. As general cognition was the strongest feature in our model to predict progression, this supports previous findings that MCI patients with deficits across multiple domains are at the highest risk for dementia [32,34].

For our VBM model, we extracted a number of gray matter atrophy subtypes that recapitulated previously reported subtypes, namely the medial temporal lobe and parietal dominant subtypes [5,28–30], which were associated strongly with a diagnosis of AD dementia. Weights for the parietal dominant and medial temporal lobe subtypes (Subtypes 1 and 6 from Figure 1b, respectively) contributed substantially to the highly predictive signature in the VBM model. The atrophy pattern of subtype 6 is spatially similar to the spread of neurofibrillary tangles in Braak stages III and IV [35], which may support previous findings that tau aggregation mediates neurodegeneration [36]. The contributions of the parietal dominant and medial temporal lobe subtypes in the VBM model are also in line with previous works, which have reported that cortical thickness and volumes of the medial temporal lobes, inferior parietal cortex, and precuneus are strong predictors of progression to dementia [7,37].

When combined with cognitive tests in the VCOG model, the structural subtypes were given marginal weights. This suggests some redundancy between atrophy and cognition, and that cognitive features have higher predictive power than structural features in the ADNI MCI sample. This conclusion is consistent with the observation that the COG model significantly outperformed the VBM model, similar to previous work [7]. Although cognitive

markers were stronger features, the VCOG model assigned large negative weights for the structural subtypes 3, which showed greater relative GMV in the temporal lobes, and 7, which showed greater relative GMV in the parietal, occipital, and temporal lobes. This means that these features were predictive of stable MCI in the VCOG model, in line with previous work showing that atrophy in these regions is predictive of progression to dementia [7,37]. Furthermore, we demonstrated that combining MRI data with cognitive markers significantly improves upon a model based on MRI features alone. This result is again in line with the literature [7,38], yet was shown for the first time for a model specifically trained for high PPV. Note that in the current study, the predictive model was trained exclusively on images acquired on 1.5T scanners from ADNI1. Good generalization to ADNI2 with 3T scanners demonstrates robustness of imaging structural subtypes across scanner makes.

The VCOG highly predictive signature might reflect a late disease stage. We looked at the ratio of early to late MCI subjects in the ADNI2 sample (note that ADNI1 did not have early MCI subjects). Of the MCI subjects identified as HPS+ by the VCOG model, 84.4% were late MCI subjects, compared to a rate of 34.9% of late MCI subjects in the entire ADNI2 MCI sample (Supplementary Figure S3). This approach may not be optimal for early detection of future cognitive decline. Training a model to classify MCI progressors and non-progressors to dementia could be done in order to capture future progressors in earlier preclinical stages (e.g. early MCI). Finally, we focused on structural MRI and neuropsychological batteries as features in our models due to their wide availability and established status as clinical tools. However, we believe adding other modalities such as PET imaging, CSF markers, functional MRI, genetic factors, or lifestyle factors could result in higher predictive power, especially at earlier preclinical stages of AD.

Conclusion

In summary, we found a subgroup of patients with MCI who share a signature of cognitive deficits and brain atrophy, that put them at very high risk to progress from MCI to AD dementia within a time span of three years. We validated the signature in two separate cohorts that contained both stable MCI patients and MCI patients who progressed to dementia. The model was able to predict progression to dementia in MCI patients with up to 93.1% PPV and up to 96.7% specificity. The signature was present in about half of all

progressors, demonstrating that gains in PPV can be made by focusing on a homogeneous, yet relatively common subgroup. Our model could potentially improve subject selection in clinical trials and identify individuals at a higher risk of AD dementia for early intervention in clinical settings.

Competing interests

The authors declare no conflicts of interest.

Acknowledgments

We would like to thank Sylvia Villeneuve, Alexa Pichet-Binette and Jacob Vogel for providing us with data to help start our preliminary analyses for this project. We would like to thank Hien Nguyen for advising us on parts of the statistical analyses.

Data collection and sharing for this project was funded by the Alzheimer's Disease Neuroimaging Initiative (ADNI) (National Institutes of Health Grant U01 AG024904) and DOD ADNI (Department of Defense award number W81XWH-12-2-0012). ADNI is funded by the National Institute on Aging, the National Institute of Biomedical Imaging and Bioengineering, and through generous contributions from the following: AbbVie, Alzheimer's Association; Alzheimer's Drug Discovery Foundation; Araclon Biotech; BioClinica, Inc.; Biogen; Bristol-Myers Squibb Company; CereSpir, Inc.; Cogstate; Eisai Inc.; Elan Pharmaceuticals, Inc.; Eli Lilly and Company; EuroImmun; F. Hoffmann-La Roche Ltd and its affiliated company Genentech, Inc.; Fujirebio; GE Healthcare; IXICO Ltd.; Janssen Alzheimer Immunotherapy Research & Development, LLC.; Johnson & Johnson Pharmaceutical Research & Development LLC.; Lumosity; Lundbeck; Merck & Co., Inc.; Meso Scale Diagnostics, LLC.; NeuroRx Research; Neurotrack Technologies; Novartis Pharmaceuticals Corporation; Pfizer Inc.; Piramal Imaging; Servier; Takeda Pharmaceutical Company; and Transition Therapeutics. The Canadian Institutes of Health Research is providing funds to support ADNI clinical sites in Canada. Private sector contributions are facilitated by the Foundation for the National Institutes of Health (www.fnih.org). The grantee organization is the Northern California Institute for Research and Education, and the study is coordinated by the Alzheimer's Therapeutic Research Institute at the University of

Southern California. ADNI data are disseminated by the Laboratory for Neuro Imaging at the University of Southern California.

The computational resources used to perform the data analysis were provided by Compute Canada (www.computecanada.org). This project was funded by NSERC grant number RN000028 and the Canadian Consortium on Neurodegeneration in Aging (CCNA, www.ccna-ccnv.ca), through a grant from the Canadian Institutes of Health Research and funding from several partners including SANOFI-ADVENTIS R&D. AT is supported by a bursary from the Centre de recherche de l'institut universitaire de gériatrie de Montréal and the Courtois foundation. CD is supported by a salary award from the Lemaire foundation and Courtois foundation. PB is supported by a salary award from Fonds de recherche du Québec -- Santé and the Courtois foundation.

References

1. Ward A, Tardiff S, Dye C, Arrighi HM. Rate of conversion from prodromal Alzheimer’s disease to Alzheimer's dementia: a systematic review of the literature. Dement Geriatr Cogn Dis Extra. 2013;3:320–32.

2. Visser P-J, Scheltens P, Verhey FRJ. Do MCI criteria in drug trials accurately identify subjects with predementia Alzheimer’s disease? J Neurol Neurosurg Psychiatry. 2005;76:1348–54.

3. Scheltens NME, Galindo-Garre F, Pijnenburg YAL, van der Vlies AE, Smits LL, Koene T, et al. The identification of cognitive subtypes in Alzheimer’s disease dementia using latent class analysis. J Neurol Neurosurg Psychiatry. 2016;87:235–43.

4. Murray ME, Graff-Radford NR, Ross OA, Petersen RC, Duara R, Dickson DW. Neuropathologically defined subtypes of Alzheimer’s disease with distinct clinical characteristics: a retrospective study. Lancet Neurol. 2011;10:785–96.

5. Hwang J, Kim CM, Jeon S, Lee JM, Hong YJ, Roh JH, et al. Prediction of Alzheimer’s disease pathophysiology based on cortical thickness patterns. Alzheimer’s & dementia (Amsterdam, Netherlands). 2016;2:58–67.

6. Dong A, Toledo JB, Honnorat N, Doshi J, Varol E, Sotiras A, et al. Heterogeneity of neuroanatomical patterns in prodromal Alzheimer’s disease: links to cognition, progression and biomarkers. Brain. 2017;140:735–47.

7. Korolev IO, Symonds LL, Bozoki AC, for the Alzheimer’s Disease Neuroimaging Initiative. Predicting Progression from Mild Cognitive Impairment to Alzheimer’s Dementia Using Clinical, MRI, and Plasma Biomarkers via Probabilistic Pattern Classification. PLoS

One. 2016;11:e0138866–25.

8. Mathotaarachchi S, Pascoal TA, Shin M, Benedet AL, Kang MS, Beaudry T, et al. Identifying incipient dementia individuals using machine learning and amyloid imaging. Neurobiol Aging. 2017;59:80–90.

9. Dansereau C, Tam A, Badhwar A, Urchs S, Orban P, Rosa-Neto P, et al. A brain signature highly predictive of future progression to Alzheimer’s dementia. arXiv preprint arXiv:1712 08058. 2017;

10. Bellec P, Lavoie-Courchesne S, Dickinson P, Lerch JP, Zijdenbos AP, Evans A. The pipeline system for Octave and Matlab (PSOM): a lightweight scripting framework and execution engine for scientific workflows. Front Neuroinform. 2012;6:7.

11. Fonov V, Evans A, Botteron K, Almli CR, McKinstry RC, Collins DL. Unbiased average age-appropriate atlases for pediatric studies. Neuroimage. 2011;54:313–27.

12. Ad-Dab’bagh Y, Einarson D, Lyttelton O, Muehlboeck JS, Mok K, Ivanov O, et al. The CIVET image-processing environment: a fully automated comprehensive pipeline for anatomical neuroimaging research. Proceedings of the 12th annual meeting of the organization for human brain mapping. 2006;2266.

13. Ashburner J. A fast diffeomorphic image registration algorithm. Neuroimage. 2007;38:95–113.

14. Orban P, Tam A, Urchs S, Savard M, Madjar C, Badhwar A, et al. Subtypes of functional brain connectivity as early markers of neurodegeneration in Alzheimer’s disease. bioRxiv. 2017;195164.

15. Zhang X, Mormino EC, Sun N, Sperling RA, Sabuncu MR, Yeo BTT, et al. Bayesian model reveals latent atrophy factors with dissociable cognitive trajectories in Alzheimer’s disease. Proc Natl Acad Sci U S A. 2016;113:E6535–44.

16. Crane PK, Carle A, Gibbons LE, Insel P, Mackin RS, Gross A, et al. Development and assessment of a composite score for memory in the Alzheimer’s Disease Neuroimaging Initiative (ADNI). Brain Imaging Behav. 2012;6:502–16.

17. Gibbons LE, Carle AC, Mackin RS, Harvey D, Mukherjee S, Insel P, et al. A composite score for executive functioning, validated in Alzheimer’s Disease Neuroimaging Initiative (ADNI) participants with baseline mild cognitive impairment. Brain Imaging Behav. 2012;6:517–27.

18. Belleville S, Fouquet C, Hudon C, Zomahoun HTV, Croteau J, Consortium for the Early Identification of Alzheimer\textquoterights disease-Quebec. Neuropsychological Measures that Predict Progression from Mild Cognitive Impairment to Alzheimer’s type dementia in Older Adults: a Systematic Review and Meta-Analysis. Neuropsychol Rev. 2017;27:328–53.

19. Pedregosa F, Varoquaux G, Gramfort A, Michel V, Thirion B, Grisel O, et al. Scikit-learn: Machine Learning in Python. J Mach Learn Res. 2011;2825–30.

20. Fan R-E, Chang K-W, Hsieh C-J, Wang X-R, Lin C-J. LIBLINEAR: A library for large

linear classification. J Mach Learn Res. 2008;9:1871–4.

21. Phipson B, Smyth GK. Permutation P-values should never be zero: calculating exact P-values when permutations are randomly drawn. Stat Appl Genet Mol Biol. 2010;9.

22. Shaw LM, Vanderstichele H, Knapik-Czajka M, Clark CM, Aisen PS, Petersen RC, et al. Cerebrospinal fluid biomarker signature in Alzheimer's disease neuroimaging initiative subjects. Ann Neurol. 2009;65:403–13.

23. Jones E, Oliphant T, Peterson P. SciPy: open source scientific tools for Python, http://www.scipy.org/. 2001;

24. Seabold S, Perktold J. Statsmodels: Econometric and statistical modeling with python. Proceedings of the 9th Python in Science Conference. SciPy society Austin; 2010.

25. Pinheiro J, Bates D, DebRoy S, Sarkar D, R Core Team. nlme: linear and nonlinear mixed effects models. R package version 3.1-117, https://CRAN.R-project.org/package=nlme. 2018;

26. Mitchell AJ, Shiri-Feshki M. Rate of progression of mild cognitive impairment to dementia - meta-analysis of 41 robust inception cohort studies. Acta Psychiatr Scand. 2009;119:252–65.

27. Gibbons LE, Carle AC, Mackin RS, Harvey D, Mukherjee S, Insel P, et al. A composite score for executive functioning, validated in Alzheimer's Disease Neuroimaging Initiative (ADNI) participants with baseline mild cognitive impairment. Brain Imaging Behav. 2012;6:517–27.

28. Noh Y, Jeon S, Lee JM, Seo SW, Kim GH, Cho H, et al. Anatomical heterogeneity of Alzheimer disease Based on cortical thickness on MRIs. Neurology. 2014;83:1936–44.

29. Park J-Y, Na HK, Kim S, Kim H, Kim HJ, Seo SW, et al. Robust Identification of Alzheimer's Disease subtypes based on cortical atrophy patterns. Sci Rep. 2017;7:43270.

30. Varol E, Sotiras A, Davatzikos C, Alzheimer's Disease Neuroimaging Initiative. HYDRA: Revealing heterogeneity of imaging and genetic patterns through a multiple max-margin discriminative analysis framework. Neuroimage. 2017;145:346–64.

31. Dickerson BC, Sperling RA, Hyman BT, Albert MS, Blacker D. Clinical prediction of Alzheimer disease dementia across the spectrum of mild cognitive impairment. Arch Gen Psychiatry. 2007;64:1443–50.

32. Tabert MH, Manly JJ, Liu X, Pelton GH, Rosenblum S, Jacobs M, et al. Neuropsychological prediction of conversion to Alzheimer disease in patients with mild cognitive impairment. Arch Gen Psychiatry. 2006;63:916–24.

33. Belleville S, Gauthier S, Lepage É, Kergoat M-J, Gilbert B. Predicting decline in mild cognitive impairment: A prospective cognitive study. Neuropsychology. 2014;28:643–52.

34. Mitchell J, Arnold R, Dawson K, Nestor PJ, Hodges JR. Outcome in subgroups of mild cognitive impairment (MCI) is highly predictable using a simple algorithm. J Neurol.

2009;256:1500–9.

35. Braak H, Braak E. Neuropathological stageing of Alzheimer-related changes. Acta Neuropathol. 1991;82:239–59.

36. Spillantini MG, Goedert M. Tau pathology and neurodegeneration. Lancet Neurol. 2013;12:609–22.

37. Eskildsen SF, Coupé P, García-Lorenzo D, Fonov V, Pruessner JC, Collins DL, et al. Prediction of Alzheimer’s disease in subjects with mild cognitive impairment from the ADNI cohort using patterns of cortical thinning. Neuroimage. 2013;65:511–21.

38. Moradi E, Pepe A, Gaser C, Huttunen H, Tohka J, Alzheimer’s Disease Neuroimaging Initiative. Machine learning framework for early MRI-based Alzheimer’s conversion prediction in MCI subjects. Neuroimage. 2015;104:398–412.

Figure Captions

Figure 1. Subtyping procedure and resulting subtypes. a) A hierarchical clustering procedure identified 7 subtypes, or subgroups, of individuals with similar patterns of grey matter topography within the ADNI1 cohort of CN and AD subjects (top). A measure of spatial similarity, called subtype weight, between a single individual's grey matter volume map and the average of a given subtype was calculated for all individuals and all subtypes (bottom). b) Maps of the 7 subtypes showing the distribution of grey matter across all voxels relative to the average. CN* and AD* denote significant associations between the subtype weights and diagnoses of cognitively normal (CN) or Alzheimer's dementia (AD) respectively.

Figure 2. Specificity, sensitivity, and positive predictive value (PPV) for the base SVM and highly predictive signature (HPS) classifiers in the classifications of a) patients with AD dementia (AD) and cognitively normal individuals (CN) and b) patients with mild cognitive impairment who progress to AD (pMCI) and stable MCI (sMCI) in ADNI1 and ADNI2. VBM represents the model trained with VBM subtypes, COG represents the model trained with baseline cognitive scores, and VCOG represents the model trained with both VBM subtypes and cognition. Significant differences are denoted by * for $p<0.05$ and ** for $p<0.001$). Positive predictive value was adjusted (PPV (adj)) for a prevalence of 33.6% pMCI in a sample of MCI subjects for both ADNI1 and ADNI2 MCI cohorts.

Figure 3. Characteristics of MCI subjects with the VCOG signature in ADNI1 and ADNI2. We show the percentage of MCI subjects who a) progressed to dementia, were b) APOE4 carriers, c) female, d) positive for Aβ measured by a cut-off of 192 pg/mL in the CSF [22], and e) positive for tau measured by a cut-off of 93 pg/mL in the CSF [22] in each classification (HPS+, Non-HPS+, and Negative). f) Age and g) cognitive trajectories, measured by the Alzheimer's Disease Assessment Scale - Cognitive subscale with 13 items (ADAS13), across the three classes. Significant differences are denoted by * for family-wise error rate-corrected $p<0.05$.

Figure 4. Coefficients of the high confidence prediction a) VCOG model, b) COG model, and c) VBM model. ADAS13=Alzheimer's Disease Assessment Scale - Cognitive, MEM=ADNI-MEM score; EXEC=ADNI-EF score, BNT=Boston Naming Test, CLOCK=clock drawing test, VBM 1-7=VBM subtype weights, GMV=mean grey matter volume, TIV=total intracranial volume.

Table 1 Click here to access/download;Table;vcog_paper_table_demog.pdf

Tables

Table 1. Demographic information for post-QC subjects in ADNI1 and ADNI2.

**ADNI1**	**CN**	**sMCI**	**pMCI**	**AD**
**N**	205	88	147	165
**Age ± SD**	76.1 ± 5.0	74.0 ± 7.6	74.3 ± 7.1	75.4 ± 7.5
**Female %**	51.7	40.9	40.8	51.5
**APOE4+ %**	27.8	37.5	68.7	65.4
**ADAS13 ± SD**	9.5 ± 4.3	14.3 ± 5.5	21.3 ± 5.3	28.6 ± 7.1
**MMSE ± SD**	29.1 ± 1.0	27.7 ± 1.7	26.7 ± 1.7	23.4 ± 2.0
**ADNI2**	**CN**	**sMCI**	**pMCI**	**AD**
**N**	188	180	55	89
**Age ± SD**	72.8 ± 6.1	70.8 ± 7.3	72.1 ± 7.1	74.4 ± 7.8
**Female %**	54.0	47.8	49.1	46.1
**APOE4+ %**	29.4	35.6	65.4	71.3
**ADAS13 ± SD**	9.1 ± 4.2	11.8 ± 5.3	21.4 ± 6.5	31.6 ± 8.7
**MMSE ± SD**	29.1 ± 1.1	28.4 ± 1.6	27.3 ± 1.9	23.1 ± 2.3

ADAS13=Alzheimer's Disease Assessment Scale - Cognitive subscale (13 items);

MMSE=Mini Mental State Examination

Figure 1

Click here to access/download;Figure;methods_subtypes_1.tiff

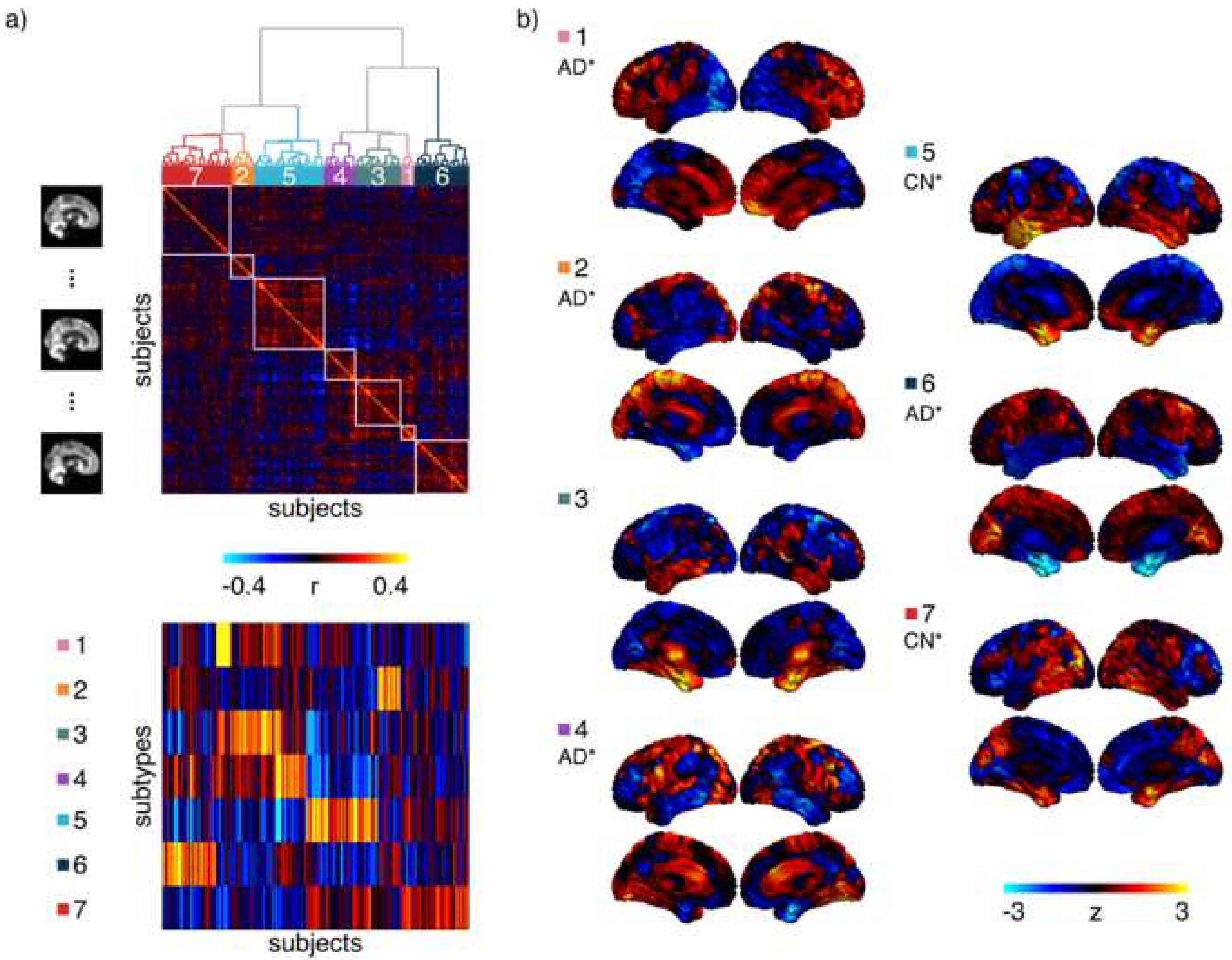

Figure 2

Click here to access/download;Figure;model_performance_mci_ad.tiff

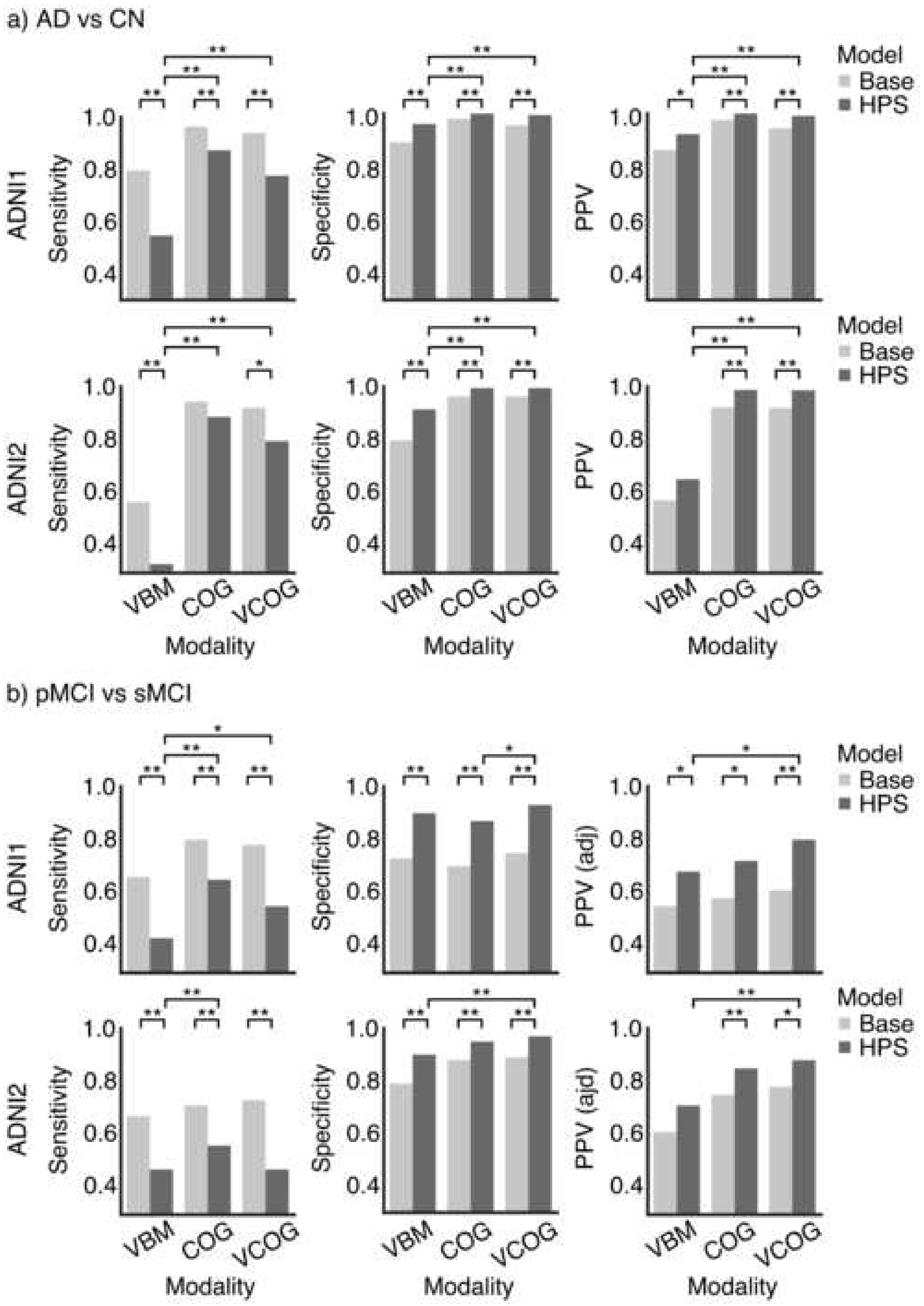

Figure 3

Click here to access/download;Figure;mci_biomarkers_hps_vcog.tiff

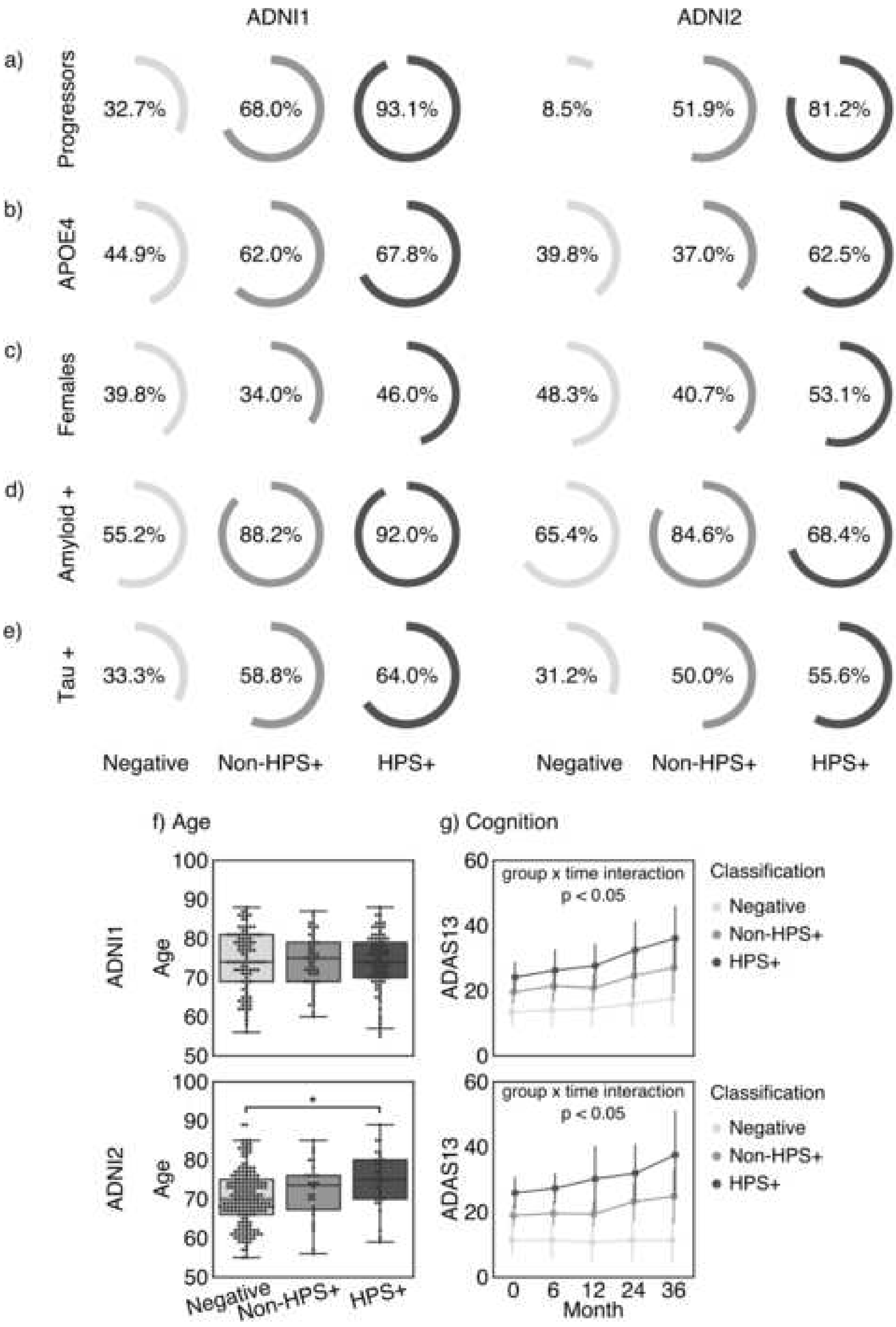

Figure 4 Click here to access/download;Figure;model_coeff_all.tiff

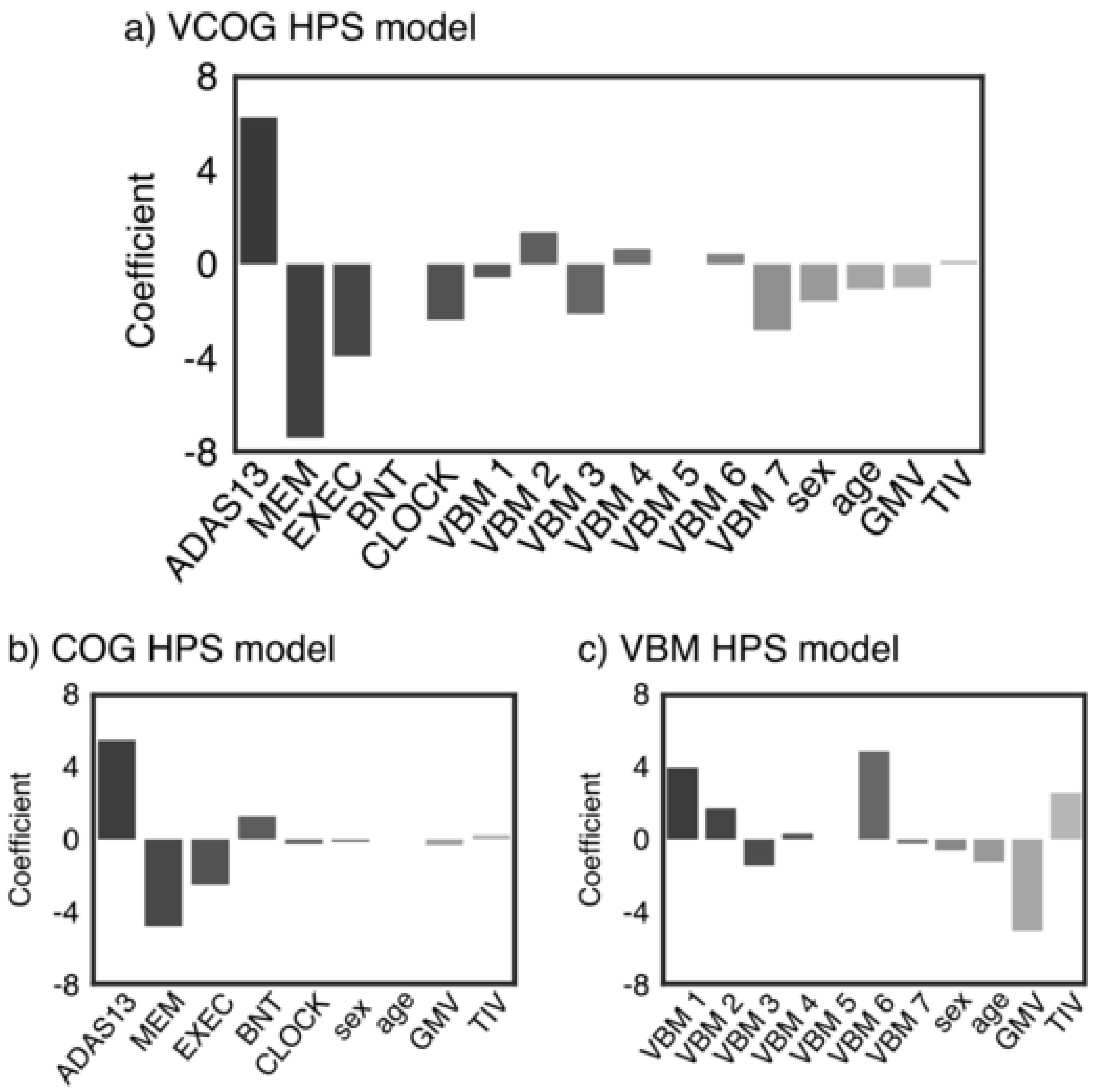

Click here to access/download
Supplementary Material
gigascience_vcog_supplemental.pdf

Letter to editor
Click here to access/download;Personal Cover;gigascience_resubmission_letter_editor.pdf

October 9, 2018

Re: Submission to GigaScience

Dear Editors,

Please find enclosed our revised manuscript titled “A highly predictive signature of cognition and brain atrophy for progression to Alzheimer's dementia” for your consideration.

We have updated our manuscript with the requested information regarding containerization and a simulated dataset.

The simulated dataset is using group-level statistics derived from the ADNI sample, but does not include any participant-level information. As such, it can be freely shared and reused. Importantly, these simulations were designed to replicate the group differences that were identified in the paper using machine learning tools. We have shared a container including all the code and dependencies used in our manuscript, and the reviewers as well as interested readers will be able to reproduce the training and testing of machine learning models from the simulated data. All the code can be executed online via the binder platform, without requiring any local installation or download.

Thank you again for your time and effort in considering our manuscript.

Best regards,

Angela Tam, PhD
On behalf of the co-authors
