# Supplementary material for: A highly predictive signature of cognition and brain atrophy for progression to Alzheimer's dementia
Source: Gigascience. 2019 May 11;8(5):giz055. doi: 10.1093/gigascience/giz055 (PMC6511068; doi:10.1093/gigascience/giz055)
Supplement: Response_to_Reviewer_Comments_Original_Submission.pdf [file giz055_response_to_reviewer_comments_original_submission.pdf]

Author's Response To Reviewer Comments

Close

Dear Editors,
Please find enclosed our revised manuscript titled "A highly predictive signature of cognition and brain atrophy for progression to Alzheimer's dementia" for your consideration.
We would like to thank the editor and the three reviewers for the constructive and insightful feedback.
We believe that this round of revision has substantially improved the paper.
Please find our responses to the reviewers' comments on the following pages.
Best regards,
Angela Tam, on behalf of the co-authors

Editor's comments:
-Overall the reviewers request more clarification in the methods/ techniques used, and also justification of the two-stage linear model chosen, over a single stage logistic regression model. They also suggest that you expand the discussion section to include more comparison of the results with other results of the proposed algorithm (i.e. PPV, sensitivity, and specificity) with that of other machine learning studies that used sMRI (or resting-state fMRI) and/or neuropsychological measures as input features.

We have followed the recommendations of the reviewers by clarifying the methods section and explaining the rationale behind the two-stage linear model. We added a collection of popular machine learning techniques as benchmark, and revised the positioning of the paper to emphasize that our contribution is to train a machine learning model in a regime of high specificity and positive positive value, rather than proposing a novel algorithm with improved overall accuracy. A more extensive survey of previously published machine learning studies that use MRI and neuropsychological measures have also been included in the discussion.

Reviewer #1 comments:
A multimodal signature of Alzheimer's dementia was first extracted using machine learning tools in the ADNI1 sample, and was comprised of cognitive deficits across multiple domains as well as atrophy in temporal, parietal and occipital regions. The authors then validated the predictive value of this signature on two MCI cohorts.

(1) How do you select the baseline of T1 scans of ADNI?

All T1-weighted MRI scans for the healthy control (CN) and Alzheimer's dementia (AD) patients that were acquired at baseline from ADNI1 and ADNI2 were included in the study. This was also the case for the patients with mild cognitive impairment (MCI), but with additional inclusion criteria. For the MCI group, each subject must have had at least 36 months of follow-up for inclusion in our study.

Please see the "Data" section under "Materials and methods" on page 5.

(2) What preprocessing techniques did you use? Did you perform any normalization technique?

Each image was linearly co-registered to MNI space using the CIVET pipeline and then segmented into grey matter, white matter, and CSF probabilistic maps with SPM12. The DARTEL toolbox was used to normalize the grey matter segmentations to a predefined grey matter template in MNI152 space. Each map was modulated to preserve the total amount of signal and then smoothed with a 8 mm isotropic Gaussian blurring kernel.

Please see the section "Structural features from voxel-based morphometry" under "Materials and methods" on page 6.

(3) Why use GMV and TIV?

TIV has been shown to have significant effects on regional grey matter volumes and has been

recommended as a variable to take into consideration for VBM analyses (Barnes, Josephine, Gerard R. Ridgway, Jonathan Bartlett, Susie M. D. Henley, Manja Lehmann, Nicola Hobbs, Matthew J. Clarkson, David G. MacManus, Sebastien Ourselin, and Nick C. Fox. 2010. "Head Size, Age and Gender Adjustment in MRI Studies: A Necessary Nuisance?" NeuroImage 53 (4): 1244–55.)

(4) Is your method a type of VBM technique?

Yes.

(5) Line 168, why use a linear support vector machine (SVM)? Did you consider to use kernel SVMs?

We have included an SVM with a RBF kernel for comparison. Please see Figure 3.

(6) Some AD detection methods could be discussed, see "Multivariate approach for Alzheimer's disease detection using stationary wavelet entropy and predator-prey particle swarm optimization" and "Single Slice based Detection for Alzheimer's disease via wavelet entropy and multilayer perceptron trained by biogeography-based optimization"

The suggested papers by reviewer #1 describe detection of patients with AD dementia from healthy controls. Since the focus of our current paper is to detect progression to AD dementia in patients with MCI from those who will remain cognitively stable, we do not think papers about classifying AD vs controls are as relevant as those that focus on progressors vs non-progressors, which we have discussed at length.

(7) How do you optimize the hyperparameters of SVM?

The hyperparameters of the SVM were optimized by a cross-validated grid search over a parameter grid. See "Prediction of high confidence AD dementia cases in ADNI1" under the "Materials and methods" section, pages 7-8.

(8) What type of t-test did you use? How did you set the confidence threshold? Did you use ANOVA?

Yes, ANOVAs were used. Tukey's HSD tests were done for the pairwise post-hoc t-tests. See "Statistical tests of association of progression, AD biomarkers, and risk factors in high confidence MCI subjects" under the "Materials and methods" section, pages 10-11.

(9) How do you combine and generate the final signature?

The third signature (VCOG) was generated by including the VBM structural subtype weights, cognitive assessment scores, mean gray matter volume, total intracranial volume, age, and sex as features into the linear SVM on ADNI1 subjects to classify AD vs controls. This process was repeated across many random subsamples, after which hit probabilities for all individual subjects were calculated. A logistic regression classifier, with L1 regularization on the coefficients, was then used to classify the subjects with 100% hit probability from everyone else. Please refer to "Prediction of high confidence AD dementia cases in ADNI1" under the "Materials and methods" section, pages 7-8.

Reviewer #2 comments:
-The aim of this manuscript was to explore whether a linear model based classifier of AD could identify MCI patients with a "highly predictive signature" of AD
and whether this represents a prodromal stage of AD by investigating how the HPS relates to genetic and phenotypic information. This is an interesting manuscript, however there are multiple opportunities for improvement, mostly with regard to justification of the 2-stage linear model, over a single stage logistic regression model.

There are two justifications for using the two-stage linear model. First, by construction, it focuses on patients for which the outcome of the stage 1 model is highly stable. Stability of prediction is valuable when selecting participants, as we would not want the inclusion criteria of a study to vary substantially based on the specific sample used to train the model. Second, by achieving stability, the two-stage model also naturally falls in a regime of high specificity. We could have used a different approach, such as thresholding the confidence score generated by the SVM, as was done by Korolev et al. (2016). But it would have required in any case the selection of an arbitrary threshold. We explain our choice of using the two-stage model in Table 1 under Objective 2b and on pages 7-8 under "Prediction of high

confidence AD dementia cases in ADNI1” in the Methods section.

-Page 6: Prediction of easy AD dementia cases in ADNI1
This section is difficult for the reader to follow. e.g. what is meant by "20% test size"? 5 fold CV?
Maybe a diagram would help to explain what is meant here.
Also this section would benefit from an explanation of the purpose of the 2-stage linear model prediction.

We used a random permutation cross-validator to split the data into 50 training and test sets, where the size of the test set was always 20% of the original sample size. This is clarified on pages 7-8 under “Prediction of high confidence AD dementia cases in ADNI1”.
We have added to this section on pages 7-8 an explanation of the two-stage model: “We then used a two-step method to select an operating point for the linear SVM to obtain a highly precise and specific classification [20]. This was done by replicating the SVM prediction via subsampling and identifying the patients with highly robust prediction outcomes, i.e. who are consistently identified as AD during testing, regardless of the training subsample and the validity of the prediction. This approach was found to lead in practice to prediction that achieve high specificity, in addition to offering a guarantee of robustness; see [20] for more information.”

-Page 6: Prediction of progression to AD dementia from the MCI stage in ADNI1
Line 191: "We re-trained our models on AD vs CN after
optimizing our hyperparameters (resampling size and resampling ratio)"
Its not clear what is meant here and also why resampling size is a hyperparameter of the model.

We have clarified the text and changed the terms resampling size and resampling ratio to number of subsamples and subsample size, respectively. We varied the number of subsamples and the subsample size to perturb the model and identify subjects that had robust outcomes during the testing phase regardless of the training subsample. Please refer to page 9 in the Methods section under “Prediction of progression to AD dementia from the MCI stage in ADNI1”.

-Page 10, Line 311: "The HPS models consistently outperformed the base SVM classifiers with respect to specificity ($p<0.001$)" Its not clear if this is a meaningful comparison (see Fig. 2 comment below)
Figure 2: Is this the most appropriate way of plotting this data? Might it be more meaningful to assess the model using the AUC of an ROC curve?
From this graph it looks as if the HPS model might be worse than the base classifier.

ROC curves have been added in a new figure (Figure 3) and AUC is reported within this figure.

-Also - naming the model HPS is confusing given the grouping of subjects into HPS, non-HPS etc.

We now refer to the HPS+ subjects as high confidence subjects and non-HPS+ as low confidence subjects.

-Page 14, Line 417 "The high specificity of our two-stage model indeed came at a cost of reduced sensitivity"
There is always a trade of between sensitivity and specificity that is not acknowledged here.

We have included a section that discusses the trade-off between sensitivity and specificity in the results: “Trade-off between sensitivity and specificity of different algorithms” on page 16. We also emphasize more this important trade-off in the abstract, introduction and the summary table of objectives, experiments and results (Table 1).

-Page 14, Line 423 "The two-stage prediction model offered the advantage of a principled approach to train the prediction model in a high-specificity regime, based on stability."
It is unclear what what "high-specificity regime" means and why the 2-stage model relates to stability.

By “high specificity regime”, we were referring to an operating point along the ROC curve where specificity is much higher compared to sensitivity. The two-stage model ensures high specificity (at a cost to sensitivity) as it selects the most robust (or stable) individuals after subsampling many times and identifying the subjects that are consistently identified as targets across the training subsamples. Please see “Prediction of high confidence AD dementia cases in ADNI1” under the Methods section on pages 7-8.

Reviewer #3 comments:
This study investigated a machine learning approach to identify high-risk MCI patients using five neuropsychological measures and structural MRI (sMRI). By combining the neuropsychological and sMRI features, the authors identified pMCI patients with 80.4% positive predictive value (PPV) in ADNI1 cohort and 87.8% PPV in ADNI2 cohort. While specificity of the proposed algorithm is high (>%95), sensitivity of the algorithm is fairly low (47.3% for ADNI2). This study addressed an important topic in Alzheimer disease which is to identify high-risk MCI patients. In addition, the manuscript was written well with clear descriptions for the methods and results. However, the novelty of this study is limited. The following comments need to be addressed.

- The emphasis of this study was to achieve a large value for PPV (and specificity) in identification of pMCI patients, but low sensitivity of the proposed algorithm was the cost of this achievement. The authors mentioned that expensive clinical trials can benefit from the proposed algorithm since false positives need to be minimized in this setting. However, this application of the proposed algorithm is arguable in that only a subset of pMCI patients (~50% of pMCI referring to ~50% sensitivity) will be identified by the algorithm and including only these extreme pMCI cases may cause a bias in results of the clinical trials.

Clinical trial inclusion criteria are typically designed to be restrictive in the aims of achieving a specific and homogenous subpopulation, therefore implementing an automatic algorithm that will maximize PPV and specificity to select individuals will help clinical trials achieve their recruitment goals in a cost and time-efficient manner.

- This study has a limited novelty which is to develop an algorithm to provide a high PPV in identification of pMCI patients, in the cost of low sensitivity. There are several studies investigated classification of pMCI and sMCI using neuroimaging (e.g. sMRI and resting-state fMRI) and/or neuropsychological measures (e.g. [Suk et al., 2014, Neuroimage 101, 569-582] and [Hojjati et al., 2018, Comput Biol Med 102, 30-39]. In fact, the authors compared PPV of their algorithm with that of only three previous studies [7-9], and two of these studies were performed by themselves. I recommend to expand this section of discussion by comparing results of the proposed algorithm (i.e. PPV, sensitivity, and specificity) with that of other machine learning studies that used sMRI (or resting-state fMRI) and/or neuropsychological measures as input features.

Other machine learning studies that used imaging and neuropsychological measures as features were indeed missing in our citations. We have expanded the list of cited works in the revision (see references #7,8,10-16). We thank the reviewer for noticing this error.

- Please add a table and summarize results of Figure 2. Please also add accuracy and AUC to this table.

We have added ROC curves as a figure (Figure 3) and AUC are now reported there.
Accuracy has now been included in Figure 2 and the results have been summarized on pages 13-15 under the sections “Prediction of AD dementia vs cognitively normal individuals”, “Identification of high confidence cases for prediction”, and “High confidence prediction of progression to AD dementia”.

Minor points:
- Line# 132: Please correct "with with" - done
- Line# 146: I recommend replacing "n subject x n subtype" to "n subject x m subtype (n=377 and m = 7)" - done
- Line#147: Please spell out VBM. - done
- Line# 185-186: "three highly predictive signatures (HPS)" in this sentence is confusing. What does the signature mean? Do you mean three models? If not, please define signature here.
Yes we meant models and have added that in for clarification.
- Figures, and in particular Figure 1, have a low quality.
We believe the figures were downsampled during the PDF build, but we changed the final format of the figures (from TIFF to PDF) for this revision.

Close
