# Supplementary material for: A highly predictive signature of cognition and brain atrophy for progression to Alzheimer's dementia
Source: Gigascience. 2019 May 11;8(5):giz055. doi: 10.1093/gigascience/giz055 (PMC6511068; doi:10.1093/gigascience/giz055)
Supplement: Response_to_Reviewer_Comments_Revision_1.pdf [file giz055_response_to_reviewer_comments_revision_1.pdf]

Author's Response To Reviewer Comments

Close

Dear Editors,

Please find enclosed our revised manuscript titled "A highly predictive signature of cognition and brain atrophy for progression to Alzheimer's dementia" for your consideration.

Thank you for the opportunity to revise our work. We agree that deep learning approaches seem poised to become the top contenders in medical imaging applications. However, based on published results, improvement in accuracy has been modest. Our simple two stage approach still allowed us to substantially improve on the precision of the diagnosis. That is not to say that the models used in our work are improvements over state-of-the-art prognosis models from a machine learning perspective. Rather, we pushed relatively standard techniques into a regime of high specificity and precision. This regime had not been explored much until this point and could prove really useful in applications such as enrichment of clinical trials. If our high precision two-stage approach were to be applied to a deep learning model with a higher baseline accuracy, we would expect an even more precise prognosis. We thus respectfully disagree with reviewer 1, as we believe this paper makes a novel and possibly important contribution to the literature. We have added these points in a new paragraph in the discussion, along with specific performance metrics from recent published deep learning studies using ADNI (please see the second paragraph of the discussion).

Best regards,
Angela Tam, on behalf of the co-authors

Close
