# Supplementary material for: A highly predictive signature of cognition and brain atrophy for progression to Alzheimer's dementia
Source: Gigascience. 2019 May 11;8(5):giz055. doi: 10.1093/gigascience/giz055 (PMC6511068; doi:10.1093/gigascience/giz055)
Supplement: Response_to_Reviewer_Comments_Revision_2.pdf [file giz055_response_to_reviewer_comments_revision_2.pdf]

Author's Response To Reviewer Comments

Close

Dear Editors,
We have revised our manuscript “A highly predictive signature of cognition and brain atrophy for progression to Alzheimer’s dementia” with the requested formatting.
Thank you for your consideration.
Best regards,
Angela Tam, on behalf of the co-authors

Close
