# Supplementary material for: A highly predictive signature of cognition and brain atrophy for progression to Alzheimer's dementia
Source: Gigascience. 2019 May 11;8(5):giz055. doi: 10.1093/gigascience/giz055 (PMC6511068; doi:10.1093/gigascience/giz055)
Supplement: Reviewer_2_Report_Original_Submission -- Hosung Kim — 12/5/2018 Reviewed [file giz055_reviewer_2_report_original_submission.pdf]

**Reviewer Report**

**Title: A highly predictive signature of cognition and brain atrophy for progression to Alzheimer's dementia**

**Version: Original Submission** **Date:** 12/5/2018

**Reviewer name: Hosung Kim**

**Reviewer Comments to Author:**

The aim of this manuscript was to explore whether a linear model based classifier of AD could identify MCI patients with a "highly predictive signature" of AD
and whether this represents a prodromal stage of AD by investigating how the HPS relates to genetic and phenotypic information. This is an interesting manuscript, however there are multiple opportunities for improvement, mostly with regard to justification of the 2-stage linear model, over a single stage logistic regression model.
Page 6: Prediction of easy AD dementia cases in ADNI1
This section is difficult for the reader to follow. e.g. what is meant by "20% test size"? 5 fold CV?
Maybe a diagram would help to explain what is meant here.
Also this section would benefit from an explanation of the purpose of the 2-stage linear model prediction.
Page 6: Prediction of progression to AD dementia from the MCI stage in ADNI1
Line 191: "We re-trained our models on AD vs CN after
optimizing our hyperparameters (resampling size and resampling ratio)"
Its not clear what is meant here and also why resampling size is a hyperparameter of the model.
Page 10, Line 311: "The HPS models consistently outperformed the base SVM classifiers with respect to specificity (p<0.001)" Its not clear if this is a meaningful comparison (see Fig. 2 comment below)
Figure 2: Is this the most appropriate way of plotting this data? Might it be more meaningful to assess the model using the AUC of an ROC curve?
From this graph it looks as if the HPS model might be worse than the base classifier.
Also - naming the model HPS is confusing given the grouping of subjects into HPS, non-HPS etc.
Page 14, Line 417 "The high specificity of our two-stage model indeed came at a cost of reduced sensitivity"
There is always a trade of between sensitivity and specificity that is not acknowledged here.
Page 14, Line 423 "The two-stage prediction model offered the advantage of a principled approach to train the prediction model in a high-specificity regime, based on stability."
It is unclear what what "high-specificity regime" means and why the 2-stage model relates to stability.

**Methods**

Are the methods appropriate to the aims of the study, are they well described, and are necessary controls included? Choose an item.

**Conclusions**

Are the conclusions adequately supported by the data shown? Choose an item.

**Reporting Standards**

Does the manuscript adhere to the journal's guidelines on [minimum standards of reporting?] Choose an item.

Choose an item.

**Statistics**

Are you able to assess all statistics in the manuscript, including the appropriateness of statistical tests used? Choose an item.

**Quality of Written English**

Please indicate the quality of language in the manuscript: Choose an item.

**Declaration of Competing Interests**

Please complete a declaration of competing interests, considering the following questions:

- Have you in the past five years received reimbursements, fees, funding, or salary from an organisation that may in any way gain or lose financially from the publication of this manuscript, either now or in the future?
- Do you hold any stocks or shares in an organisation that may in any way gain or lose financially from the publication of this manuscript, either now or in the future?
- Do you hold or are you currently applying for any patents relating to the content of the manuscript?
- Have you received reimbursements, fees, funding, or salary from an organization that holds or has applied for patents relating to the content of the manuscript?
- Do you have any other financial competing interests?
- Do you have any non-financial competing interests in relation to this paper?

If you can answer no to all of the above, write 'I declare that I have no competing interests' below. If your reply is yes to any, please give details below.

I declare that I have no competing interests

I agree to the open peer review policy of the journal. I understand that my name will be included on my report to the authors and, if the manuscript is accepted for publication, my named report including any attachments I upload will be posted on the website along with the authors' responses. I agree for my

report to be made available under an Open Access Creative Commons CC-BY license (http://creativecommons.org/licenses/by/4.0/). I understand that any comments which I do not wish to be included in my named report can be included as confidential comments to the editors, which will not be published.

I agree to the open peer review policy of the journal

To further support our reviewers, we have joined with Publons, where you can gain additional credit to further highlight your hard work (see: https://publons.com/journal/530/gigascience). On publication of this paper, your review will be automatically added to Publons, you can then choose whether or not to claim your Publons credit. I understand this statement.

Yes Choose an item.
