# Supplementary material for: A highly predictive signature of cognition and brain atrophy for progression to Alzheimer's dementia
Source: Gigascience. 2019 May 11;8(5):giz055. doi: 10.1093/gigascience/giz055 (PMC6511068; doi:10.1093/gigascience/giz055)
Supplement: Reviewer_3_Report_Original_Submission -- Abbas Babajani-Feremi — 12/21/2018 Reviewed [file giz055_reviewer_3_report_original_submission.pdf]

Reviewer Report

Title: A highly predictive signature of cognition and brain atrophy for progression to Alzheimer's dementia

Version: Original Submission **Date:** 12/21/2018

Reviewer name: Abbas Babajani-Feremi

Reviewer Comments to Author:

This study investigated a machine learning approach to identify high-risk MCI patients using five neuropsychological measures and structural MRI (sMRI). By combining the neuropsychological and sMRI features, the authors identified pMCI patients with 80.4% positive predictive value (PPV) in ADNI1 cohort and 87.8% PPV in ADNI2 cohort. While specificity of the proposed algorithm is high (>%95), sensitivity of the algorithm is fairly low (47.3% for ADNI2). This study addressed an important topic in Alzheimer disease which is to identify high-risk MCI patients. In addition, the manuscript was written well with clear descriptions for the methods and results. However, the novelty of this study is limited. The following comments need to be addressed.

- The emphasis of this study was to achieve a large value for PPV (and specificity) in identification of pMCI patients, but low sensitivity of the proposed algorithm was the cost of this achievement. The authors mentioned that expensive clinical trials can benefit from the proposed algorithm since false positives need to be minimized in this setting. However, this application of the proposed algorithm is arguable in that only a subset of pMCI patients (~50% of pMCI referring to ~50% sensitivity) will be identified by the algorithm and including only these extreme pMCI cases may cause a bias in results of the clinical trials.
- This study has a limited novelty which is to develop an algorithm to provide a high PPV in identification of pMCI patients, in the cost of low sensitivity. There are several studies investigated classification of pMCI and sMCI using neuroimaging (e.g. sMRI and resting-state fMRI) and/or neuropsychological measures (e.g. [Suk et al., 2014, Neuroimage 101, 569-582] and [Hojjati et al., 2018, Comput Biol Med 102, 30-39]. In fact, the authors compared PPV of their algorithm with that of only three previous studies [7-9], and two of these studies were performed by themselves. I recommend to expand this section of discussion by comparing results of the proposed algorithm (i.e. PPV, sensitivity, and specificity) with that of other machine learning studies that used sMRI (or resting-state fMRI) and/or neuropsychological measures as input features.
- Please add a table and summarize results of Figure 2. Please also add accuracy and AUC to this table.

Minor points:

- Line# 132: Please correct "with with"
- Line# 146: I recommend replacing "n subject x n subtype" to "n subject x m subtype (n=377 and m = 7)"
- Line#147: Please spell out VBM.
- Line# 185-186: "three highly predictive signatures (HPS)" in this sentence is confusing. What does the signature mean? Do you mean three models? If not, please define signature here.
- Figures, and in particular Figure 1, have a low quality.

Methods

Are the methods appropriate to the aims of the study, are they well described, and are necessary controls included? Choose an item.

Conclusions

Are the conclusions adequately supported by the data shown? Choose an item.

Reporting Standards

Does the manuscript adhere to the journal's guidelines on [minimum standards of reporting?] Choose an item.

Choose an item.

Statistics

Are you able to assess all statistics in the manuscript, including the appropriateness of statistical tests used? Choose an item.

Quality of Written English

Please indicate the quality of language in the manuscript: Choose an item.

Declaration of Competing Interests

Please complete a declaration of competing interests, considering the following questions:

- Have you in the past five years received reimbursements, fees, funding, or salary from an organisation that may in any way gain or lose financially from the publication of this manuscript, either now or in the future?
- Do you hold any stocks or shares in an organisation that may in any way gain or lose financially from the publication of this manuscript, either now or in the future?
- Do you hold or are you currently applying for any patents relating to the content of the manuscript?
- Have you received reimbursements, fees, funding, or salary from an organization that holds or has applied for patents relating to the content of the manuscript?
- Do you have any other financial competing interests?
- Do you have any non-financial competing interests in relation to this paper?

If you can answer no to all of the above, write 'I declare that I have no competing interests' below. If your reply is yes to any, please give details below.

None

I agree to the open peer review policy of the journal. I understand that my name will be included on my report to the authors and, if the manuscript is accepted for publication, my named report including any attachments I upload will be posted on the website along with the authors' responses. I agree for my report to be made available under an Open Access Creative Commons CC-BY license (http://creativecommons.org/licenses/by/4.0/). I understand that any comments which I do not wish to be included in my named report can be included as confidential comments to the editors, which will not be published.

I agree to the open peer review policy of the journal

To further support our reviewers, we have joined with Publons, where you can gain additional credit to further highlight your hard work (see: https://publons.com/journal/530/gigascience). On publication of this paper, your review will be automatically added to Publons, you can then choose whether or not to claim your Publons credit. I understand this statement.

Yes Choose an item.
